# Genome-Wide Association Identifies Candidate Genes for Salt Tolerance in Soybean at Emergence and Seedling Stages

**DOI:** 10.3390/biology15161409

**Published:** 2026-08-17

**Authors:** Xiaojian Luo, Yuemei Ji, Jiangyuan Xu, Yongzhe Gu, Jun Wang, Zhangxiong Liu, Lijuan Qiu

**Affiliations:** 1 State Key Laboratory of Crop Gene Resources and Breeding, National Key Facility for Gene Resources and Genetic Improvement, Key Laboratory of Crop Germplasm Utilization of Ministry of Agriculture, Institute of Crop Sciences, Chinese Academy of Agricultural Sciences, Beijing 100081, China; 2 Institute of Crops, Ningxia Academy of Agricultural and Forestry Sciences, Yinchuan 750002, China; 3 The Shennong Laboratory, Zhengzhou 450002, China

**Keywords:** soybean, salt tolerance, genome-wide association study (GWAS), candidate genes

## Abstract

Salt stress is an abiotic constraint on crop production, impairing growth while reducing yield and quality. As a source of edible oil and protein, soybean is particularly vulnerable to salt stress during emergence and seedling establishment, so the genetic basis of salt tolerance bears directly on productive cultivation. Here, a natural population of 256 soybean accessions was phenotyped for salt tolerance at emergence and seedling stages and genotyped using the Zhongdouxin No.1 (ZDX1) single nucleotide polymorphism (SNP) array. Genome-wide association analysis identified 60 salt tolerance-associated SNPs consolidated into 19 quantitative trait loci (QTL), 16 associated with emergence-stage and 3 with seedling-stage salt tolerance. Five QTLs mapped to chromosomal regions harboring previously reported stress-tolerance genes, including GmSALT3, approximately 192 kb from qSTG-SSB-03. Four putative candidate genes were prioritized: *Glyma.10G040000*, encoding a glutathione S-transferase (GST), was identified at the emergence stage, while *Glyma.10G148700*, *Glyma.10G149200*, and *Glyma.10G149600*, encoding a calmodulin-binding protein (CaM), drought-induced protein 19 (Di19), and a protein phosphatase 2C (PP2C), respectively, were identified at the seedling stage, all with reported roles in salt-stress responses.

## Simple Summary

Soil salinization is an expanding threat to soybean production worldwide. Resolving the genetic basis of salt tolerance at early growth stages supports the development of resilient cultivars. In this study, we evaluated 256 soybean accessions for salt tolerance at emergence and seedling stages and conducted genome-wide association analysis (GWAS) using a high-density single nucleotide polymorphism (SNP) array. We identified 19 genomic regions (quantitative trait loci, QTLs) associated with salt tolerance, including 16 at the emergence stage and 3 at the seedling stage, along with four putative candidate genes with predicted functions in stress responses. Salt tolerance at the two developmental stages was weakly correlated, indicating that tolerance is stage-specific and that at each stage requires separate selection. These findings provide genetic targets for breeding salt-tolerant soybean varieties.

## 1. Introduction

Soybean (*Glycine max* [L.] Merr.) is a food and oilseed crop whose stable production underpins global food security and agricultural economies [1]. Soil salinization limits soybean growth, development, and yield by disrupting cellular homeostasis through ion toxicity, osmotic stress, and oxidative damage, suppressing germination, root development, photosynthetic efficiency, and biomass accumulation [2,3]. Globally, an estimated 950 million hectares are affected by salinity, including roughly 20% of the 230 million hectares of irrigated land, with the affected area expanding at approximately 10% annually [4,5,6,7]. Developing salt-tolerant cultivars is therefore among the lowest-cost strategies to address this challenge [8].

Salt tolerance in soybean is a quantitative trait governed by multiple genes operating through a complex regulatory network [9]. Tolerance is developmentally stage-specific: an accession tolerant at germination or seedling establishment does not necessarily perform well at maturity, and tolerance across stages is often uncorrelated [10,11,12,13]. Furthermore, salt stress during early development suppresses canopy establishment and vegetative growth, with cascading effects on subsequent stages that ultimately reduce both seed yield and protein content [14,15,16]. Mapping genetic loci underlying salt tolerance specifically at emergence and seedling stages therefore supports the breeding of improved cultivars [17].

Early efforts to map soybean salt tolerance relied on biparental populations. Lee et al. [18] used an F_2:5_ population from a cross between the salt-tolerant cultivar S-100 and the salt-sensitive cultivar Tokyo. This study mapped a major seedling-stage quantitative trait loci (QTL) to a 3.6 cM interval flanked by simple sequence repeat (SSR) markers Sat_091 and Satt237 on chromosome 3. This locus was detected again in subsequent studies [19,20,21,22]. In 2014, Qi et al. [23] and Guan et al. [24] independently cloned the underlying gene, *Glyma03g32900*, from wild and cultivated soybean, naming it *GmCHX1* and *GmSALT3*, respectively; the encoded cation Na^+^/H^+^ transporter limits Na^+^ accumulation in seedling leaves, markedly improving salt tolerance. Do et al. [20] crossed salt-tolerant Fiskeby III with moderately salt-sensitive Williams 82, generating 132 F_2_ families, and mapped a QTL on chromosome 13 linked to leaf sodium concentration (LSC). More recently, Li et al. [25] used a recombinant inbred line (RIL) population derived from Williams 82 × PI483460B to map *qSalt_Gm18* on chromosome 18, alongside *qSalt_Gm03*, which co-localizes with *GmCHX1* on chromosome 3. To date, 68 soybean salt tolerance QTLs identified from biparental populations have been cataloged in SoyBase (https://www.soybase.org).

Relative to biparental linkage mapping, genome-wide association studies (GWASs) exploit linkage disequilibrium (LD) within natural populations with distinct advantages: broader allelic diversity, finer mapping resolution, and no requirement to construct segregating populations [26,27,28]. High-density single nucleotide polymorphism (SNP)-based GWAS has been widely deployed across crops, including rice [29], cowpea [30], rapeseed [31], cotton [32], alfalfa [33], and sesame [34], and is now routine in plant molecular breeding [35]. Recent GWASs have expanded our understanding of salt tolerance loci in soybean. Kan et al. [36] applied GWAS to 191 landraces at the germination stage, identifying one significant SNP on chromosome 9 associated with the germination index ratio and seven SNPs on chromosomes 2, 3, 9, 12, and 13 associated with the germination rate ratio. Wang et al. [37] detected 1841 significant SNPs associated with germination-stage salt tolerance, developed Kompetitive Allele-Specific PCR (KASP) markers, and validated three candidate genes with elevated expression under salt stress via quantitative real-time polymerase chain reaction (qRT-PCR). Do et al. [38] identified an additional seedling-stage salt tolerance locus on chromosome 8 beyond the *GmSALT3* region on chromosome 3 through high-resolution GWAS. Dong et al. [39] identified a major salt tolerance locus controlled by *E2*, a homolog of *Arabidopsis GIGANTEA* (*GI*), using leaf chlorosis as the phenotypic indicator; *E2* knockout enhanced tolerance by promoting peroxidase activity and reducing reactive oxygen species (ROS) accumulation under salt stress. Pruthi et al. [40] conducted association mapping across cultivated and wild soybean accessions, identifying candidate genes such as *GmKUP6* and *GmWRKY33*.

Despite these advances, genetic studies of salt tolerance specifically at the emergence stage remain limited. In this study, a natural population of 256 soybean accessions was evaluated for salt tolerance at both emergence and seedling stages under controlled salt stress and genotyped with a high-density SNP array. GWAS was then conducted to identify associated loci and candidate genes, providing a resource for gene cloning and marker-assisted selection.

## 2. Materials and Methods

### 2.1. Materials

A total of 256 soybean accessions were used, comprising 255 Chinese accessions and one accession of foreign origin (Table S1). The salt-tolerant accession Zhonghuang 39 and the salt-sensitive accession NY27-38 served as controls throughout emergence-stage evaluations.

### 2.2. Salt Stress Treatment and Tolerance Assessment at the Emergence Stage

For each accession, 60 mature, uniformly sized seeds of consistent shape and color were selected. Small pots (7 × 7 × 8 cm) were filled with vermiculite to 2 cm below the rim, sown with 10 seeds each, and topped with vermiculite to the rim. Every 24 pots were arranged in a large container (46 × 32 × 10 cm). For salt treatment, 6 L of 150 mmol/L NaCl solution was added to each large container; after 5 min of saturation, the small pots were transferred to a clean container. Subsequently, 3 L of reverse-osmosis water (RO water) was replenished every 3 days. Once the vermiculite reached its maximum water-holding capacity, the pots were immediately moved to a fresh container to prevent salt leaching. Control treatments were conducted identically, substituting 6 L of RO water for the NaCl solution. All treatments were performed in triplicate. 

**Figure 1:**
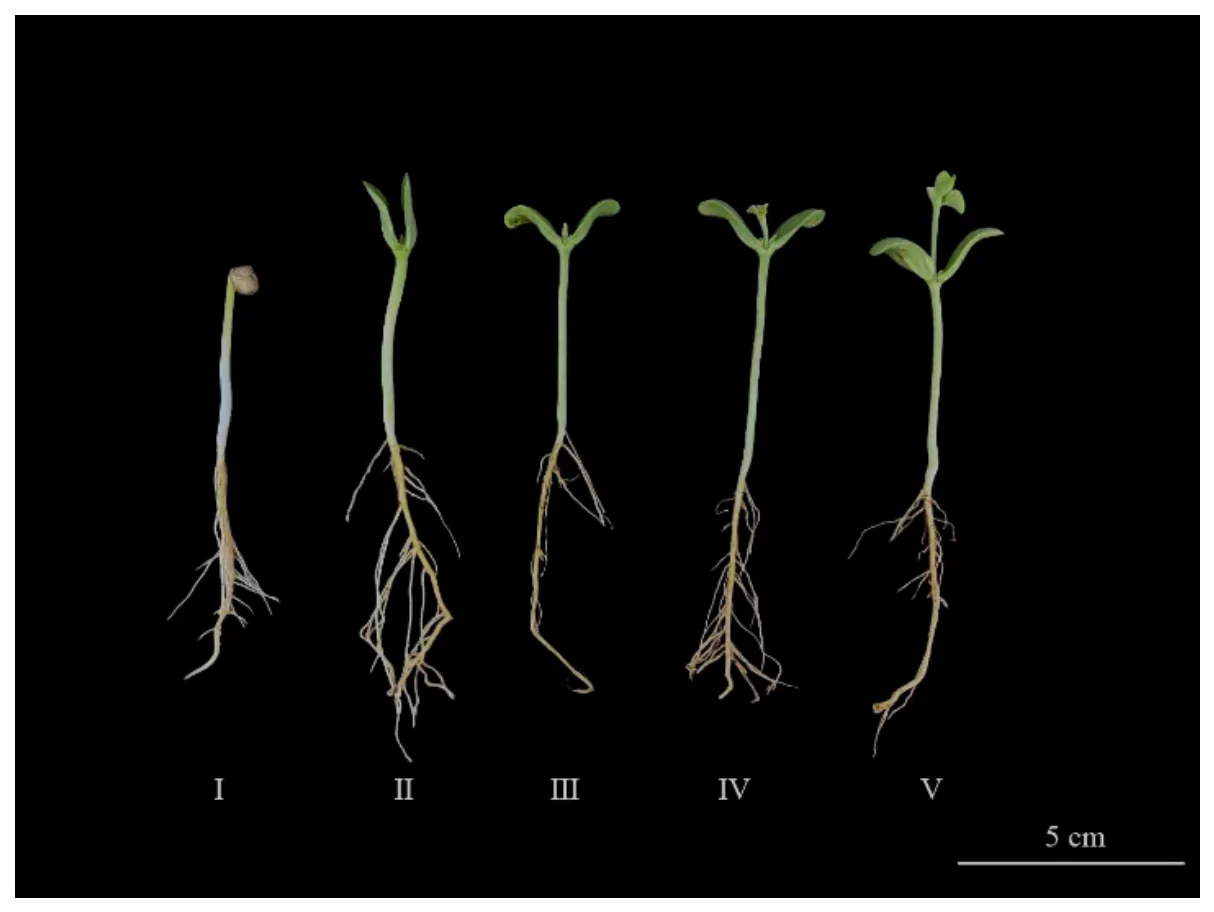
Individual-plant classification criteria for salt tolerance at the emergence stage.

Emergence was scored daily from the appearance of the first seedling, defined as cotyledon protrusion above the vermiculite surface, until NY27-38 displayed salt-injury symptoms (severely suppressed emergence and failure of cotyledon expansion). At the same time, Zhonghuang 39 remained unaffected, with normal emergence and cotyledon expansion. At this endpoint, the number of fully established seedlings (those with expanded cotyledons and leaves) was recorded. Salt tolerance phenotypes were assessed using an individual-plant classification scheme (Figure 1; Table S2) according to Liu et al. [41], from which the salt tolerance index (SI) was calculated. Emergence-stage salt tolerance grade (STG-SI) was assigned based on the mean SI across three replicates according to Table S2 (Figure S1). The SI was calculated as:

$$\mathrm{SI}=\sum (I\times N_{i})/(10\times 5)$$

where *I* is the individual-plant category value, *N*_i_ is the number of plants in category *I*, 10 is the number of seeds sown per pot, and 5 is the maximum category value. The salt tolerance coefficient (ST) was the percentage of emerged seedlings under salt stress relative to the control. 

### 2.3. Salt Stress Treatment and Tolerance Assessment at the Seedling Stage

Seed preparation and sowing for the seedling-stage assay followed the same protocol as described for the emergence stage. After sowing, 6 L of RO water was added to each large container. After 5 min of saturation, the pots were transferred to a clean container, and 3 L of RO water was replenished every 3 days using the same procedure. Once unifoliate leaves were fully expanded (approximately 10 days after sowing), salt treatment was initiated by adding 3 L of 200 mmol/L NaCl solution to each container. Following saturation, pots were moved to clean containers, and this treatment was repeated on days 13 and 16. Five days after the final salt application, leaf salt-injury symptoms were scored in each replicate according to a five-grade scale (Table S3; Figure S2) following Liu [42]: grade 1, highly tolerant; grade 2, tolerant; grade 3, moderately tolerant; grade 4, sensitive; grade 5, highly sensitive. The mean grade across three replicates was calculated to assign the final seedling-stage salt tolerance grade (STG-SS), with means between consecutive integers rounded to the higher grade.

### 2.4. Phenotypic Data Processing and Analysis

Descriptive statistics and normality testing were performed in SPSS (v25.0, IBM), and one-way ANOVA was performed in R (v4.4.2). The emergence-stage salt tolerance coefficient (ST) and salt tolerance index (SI) were standardized by rank-based inverse normal transformation (INT) following Zachary et al. [43], yielding the transformed traits ST-INT and SI-INT, respectively.

For ordinal traits with small, skewed distributions, binarization is a recognized strategy in GWAS that sharpens contrast between phenotypic extremes and increases power to detect association signals [44]. Accordingly, STG-SI and STG-SS were each binarized: accessions scoring below 3 were classified as strongly tolerant (coded 1) and those scoring 3 or above were weakly tolerant (coded 0), producing binary traits STG-SIB and STG-SSB, respectively. Pearson correlation coefficients among ST-INT, SI-INT, STG-SIB, and STG-SSB were computed using the psych package (v2.6.3) in R (v4.4.2) and visualized with the ggpairs function in the GGally package (v2.4.0).

### 2.5. Quality Control of Genotype Data

Genomic DNA was extracted from all 256 accessions and genotyped using a ZDX1 SNP array, co-developed by the Institute of Crop Sciences, Chinese Academy of Agricultural Sciences, and Beijing Compass Biotechnology Co., Ltd. (Beijing, China) SNP quality control followed Sun et al. [45], with PLINK (v1.9) used to remove SNPs with genotype missing rate > 0.4 or minor allele frequency < 0.05.

### 2.6. Linkage Disequilibrium Estimation

Linkage disequilibrium (LD) decay was estimated using PopLDdecay (v3.31; BGI Genomics) and visualized in R (v4.4.2) with ggplot2 (v3.4.3). The physical distance at which r^2^ declined to half of its maximum value (i.e., from an initial r^2^ of approximately 1.0 to r^2^ ≈ 0.5) was taken as the LD decay distance for this population.

### 2.7. Population Structure and Kinship Analysis

K-means clustering was conducted using the kmeans function in R (v4.4.2), with the optimal number of subpopulations identified by the elbow method, plotting the sum of squared errors (SSEs) for K = 2–10 and selecting the K at which SSE decline flattened. Principal component analysis (PCA) was conducted with the prcomp function in R (v4.4.2). A neighbor-joining (NJ) phylogenetic tree was constructed from pairwise genetic distance matrices using the ape (v5.8-1) and ggtree (v3.16.0) packages. Population structure was additionally estimated with ADMIXTURE across K = 2–8, with the optimal K selected by minimizing cross-validation (CV) error. The final subpopulation assignment integrated results from K-means clustering, PCA, the NJ tree, and ADMIXTURE. A kinship matrix was computed from SNP data using GAPIT (v3.5; http://zzlab.net/GAPIT, accessed on 14 September 2025), and relatedness within and between subpopulations was visualized as a heatmap.

### 2.8. Genome-Wide Association Analysis (GWAS)

GWAS was conducted using the GAPIT platform (v3.5; http://zzlab.net/GAPIT, accessed on 14 September 2025) with seven models, including GLM, MLM, CMLM, MLMM, SUPER, FarmCPU, and BLINK, with population structure and kinship coefficients included as covariates to control false positives. Association loci were declared at a suggestive threshold of −log_10_(*P*) ≥ 4. Only those detected by at least three of the seven models were retained to minimize false positives, and results were visualized using ggplot2 (v3.4.3) in R (v4.4.2). Adjacent significant SNPs on the same chromosome, with pairwise distances within the LD decay distance (~50 kb), were consolidated into a single QTL. The SNP with the highest −log_10_(*P*) value within each QTL was the peak SNP.

All seven models implemented in GAPIT are based on linear or linear mixed model frameworks. For the binary traits (STG-SIB and STG-SSB), linear models were applied to the 0/1 coded variables. Although logistic models are theoretically more appropriate for binary outcomes, linear models have been widely adopted in plant GWAS for binary and categorical traits and perform adequately when category distributions are reasonably balanced, as in the present study (60.16% vs. 39.84% for STG-SIB; 62.50% vs. 37.50% for STG-SSB). Concordant QTL signals from the continuous emergence-stage traits (ST-INT and SI-INT) support the binary-trait results at that stage; no continuous trait was measured at the seedling stage, so STG-SSB carries no equivalent support.

### 2.9. Candidate Gene Identification

Haplotype blocks around significant SNPs were identified using LDBlockShow (v1.40). For each stable LD block, the SNP with the highest −log_10_(*P*) value was the peak SNP, and protein-coding genes within the haplotype block boundaries extended by 50 kb on either side were identified as candidate genes based on the population-specific LD decay distance. Tissue-level expression profiles of candidate genes were examined using the Soybean eFP Browser (https://bar.utoronto.ca/efp_soybean/, accessed on 25 June 2026). Putative candidate genes were prioritized based on functional annotation for stress-related roles, expression in relevant tissues, and published evidence of involvement in salt tolerance.

## 3. Results

### 3.1. Phenotypic Evaluation of Salt Tolerance at Emergence and Seedling Stages 

At the emergence stage, the mean emergence rate under control conditions was 0.93 (range: 0.4–1.0). Salt stress reduced this to a mean of 0.85 (range: 0–1.0), a difference supported by one-way ANOVA (*p* < 0.0001) (Figure S3).

At the emergence stage, mean ST was 89.32 (range: 33.33–100) and mean SI was 0.62 (range: 0.09–1.00) (Table 1). Both traits deviated markedly from normality: ST was left-skewed (skewness = −1.90) with a leptokurtic distribution (kurtosis = 4.60), while SI exhibited mild left skewness (skewness = −0.50). Rank-based inverse normal transformation was applied to both, and the resulting ST-INT and SI-INT approximated normal distributions, with skewness and kurtosis near zero, means near zero, and variance near one (Table 1; Figure S4A,B). STG-SI and STG-SS were both right-skewed (skewness = 0.52 and 0.66, respectively; Table 1) and were thus binarized, yielding STG-SIB and STG-SSB with strongly tolerant proportions of 60.16% and 62.50%, sufficiently balanced for association analysis (Figure S4C,D). All four transformed traits were carried forward for GWAS.

### 3.2. Correlation Analysis

**Figure 2:**
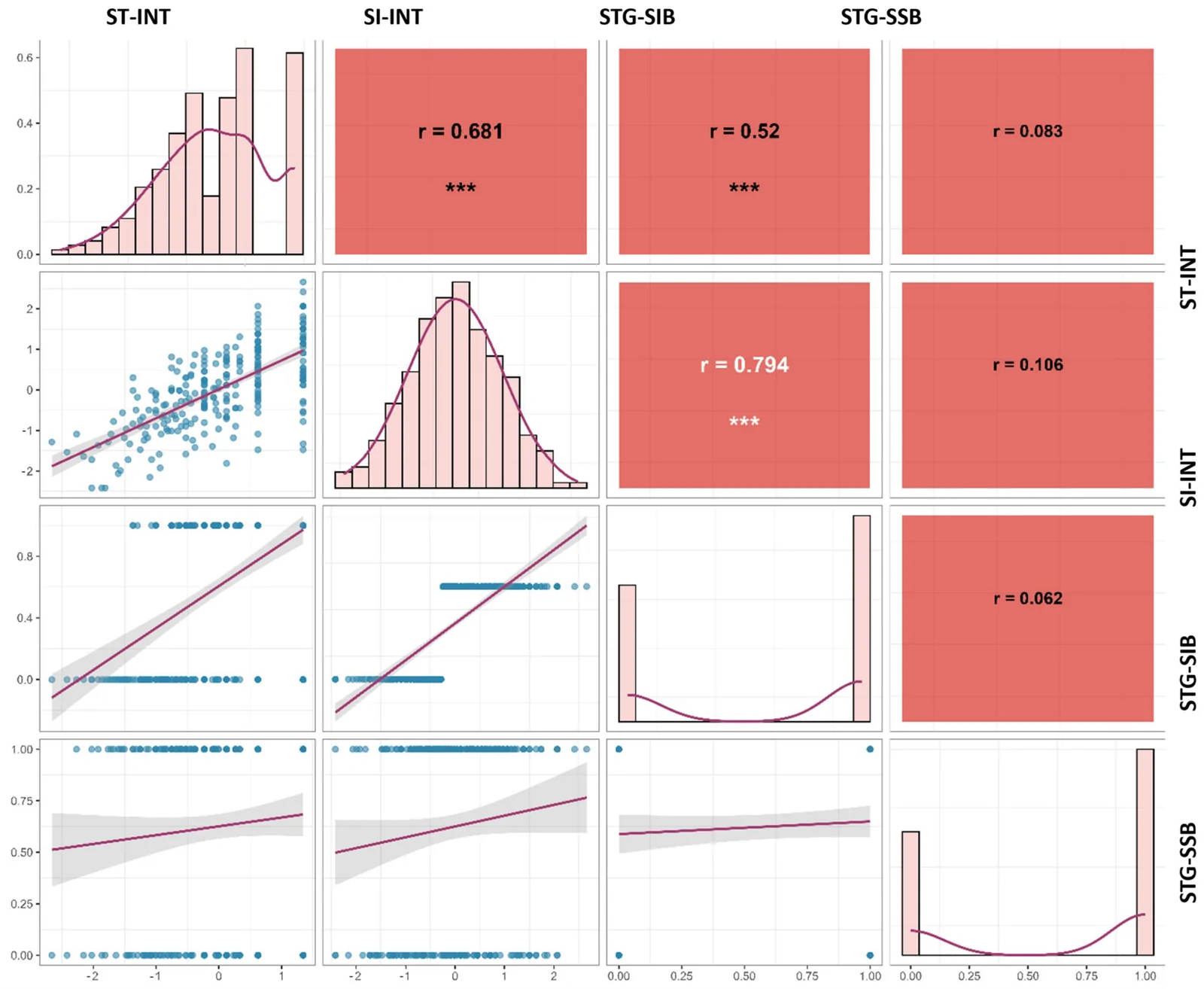
Phenotypic trait correlation matrix. Upper triangle: Pearson correlation coefficients with significance indicators (*** *p* < 0.001); diagonal: frequency distribution histograms for ST-INT, SI-INT, STG-SIB, and STG-SSB; lower triangle: scatter plots with fitted regression lines. ST-INT is the salt tolerance coefficient after inverse normal transformation at the emergence stage; SI-INT is the salt tolerance index after inverse normal transformation at the emergence stage; STG-SIB is the salt tolerance index grades after binary conversion at the emergence stage; STG-SSB is the salt tolerance grades following binary conversion at the seedling stage.

Pearson correlation analysis among ST-INT, SI-INT, STG-SIB, and STG-SSB showed positive associations among all three emergence-stage traits (Figure 2): ST-INT vs. SI-INT, *r* = 0.681; ST-INT vs. STG-SIB, *r* = 0.520; SI-INT vs. STG-SIB, *r* = 0.794, all significant. In contrast, the seedling-stage trait STG-SSB showed negligible correlations with STG-SIB (*r* = 0.062), ST-INT (*r* = 0.083), and SI-INT (*r* = 0.106), indicating that salt tolerance at emergence and at the seedling stage are weakly correlated in this population, likely reflecting distinct aspects of the salt stress response.

### 3.3. Genotype Data Quality Control

**Figure 3:**
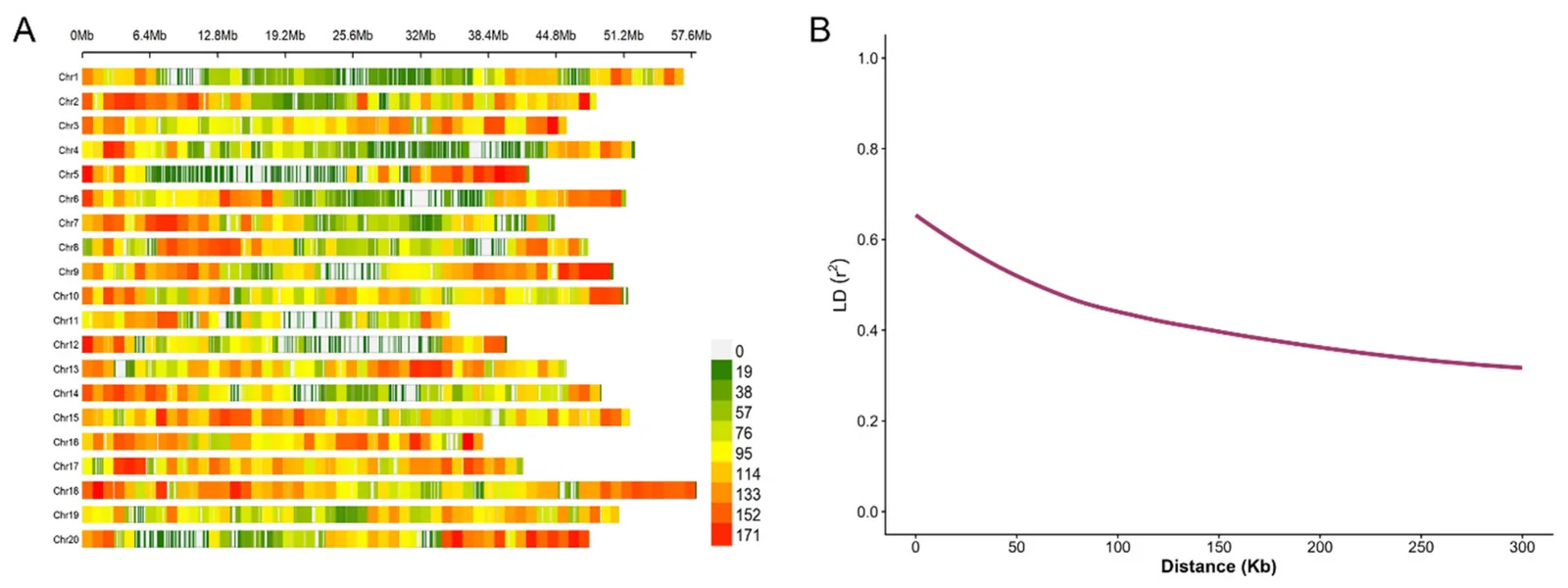
Genomic distribution of SNP markers and linkage disequilibrium in the 256-accession panel. (**A**) Density of the 89,361 SNPs across the 20 soybean chromosomes in 1 Mb windows; (**B**) LD decay curve showing the decline in r^2^ with increasing physical distance.

ZDX1 SNP array genotyping across all 256 accessions initially yielded 159,064 SNPs (Figure 3A). Following PLINK-based filtering for missing rate and minor allele frequency, 89,361 SNPs were retained for GWAS.

### 3.4. Linkage Disequilibrium 

PopLDdecay analysis showed that r^2^ declined with increasing physical distance, from an initial r^2^ of approximately 1.0 to half of its maximum value (r^2^ ≈ 0.5) at approximately 50 kb (Figure 3B), which was defined as the LD decay distance for candidate gene interval definition.

### 3.5. Population Structure Analysis Based on Clustering, Phylogeny, and Principal Components

**Figure 4:**
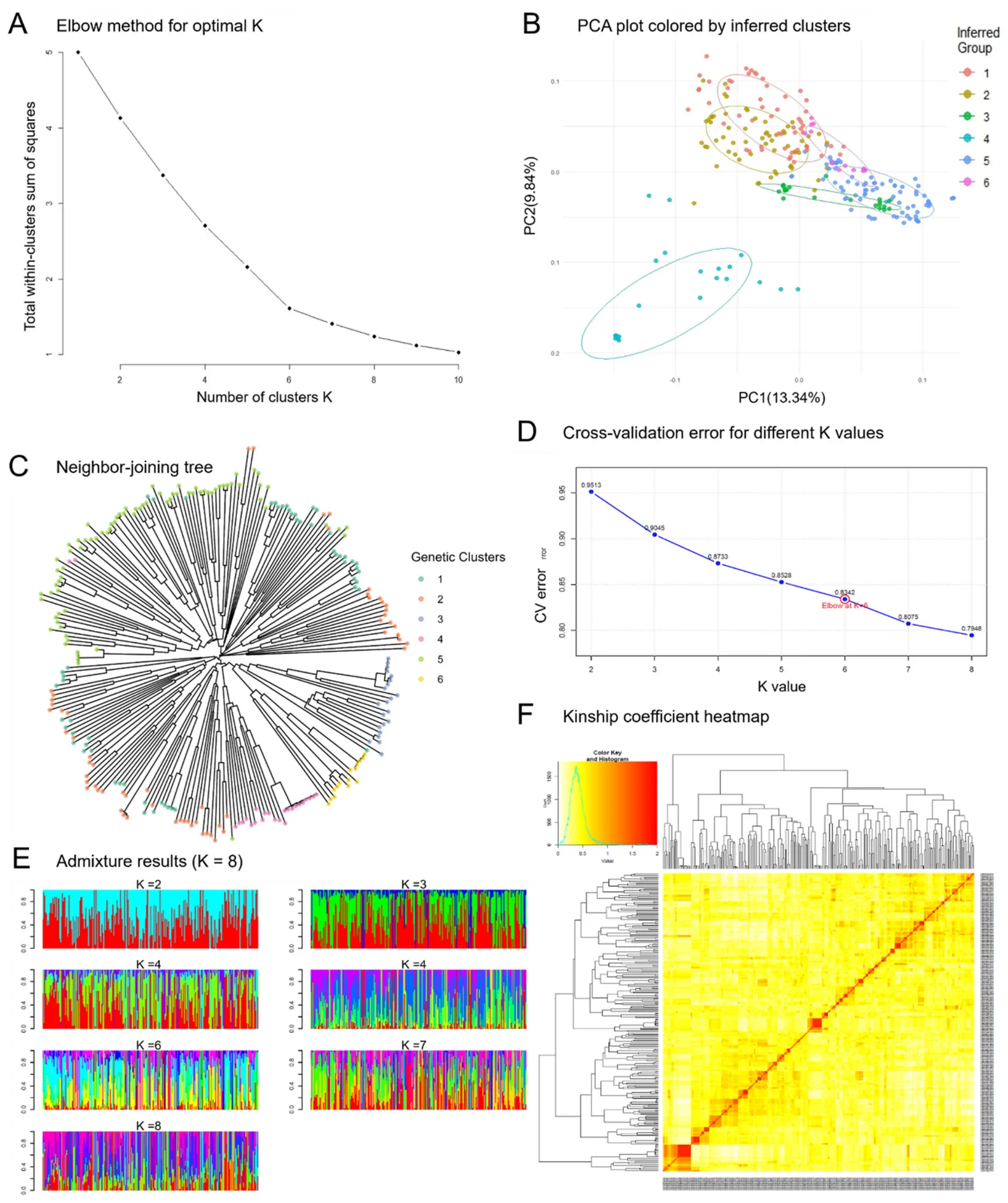
Genetic structure and relatedness of 256 soybean accessions. (**A**) Elbow plot from K-means clustering showing the inflection point at K = 6; (**B**) PCA scatter plot (PC1 vs. PC2) with accessions colored by the six inferred subpopulations; (**C**) Neighbor-joining phylogenetic tree with branches colored by subpopulation assignment; (**D**) Cross-validation error curves across K = 2–8 used to guide ADMIXTURE model selection; (**E**) ADMIXTURE ancestry plots for K = 2–8, where each vertical bar represents one accession and colored segments denote the proportional ancestry contribution from each inferred source population; (**F**) Kinship heatmap from hierarchical clustering of all 256 accessions, with darker shading indicating closer relatedness.

Four complementary methods were employed to characterize the structure of this population. K-means clustering with the elbow method showed an inflection point in the within-cluster sum of squares at K = 6, taken as the clustering solution (Figure 4A). PCA was consistent with this result, showing genetic stratification in which PC1 and PC2 explained 13.34% and 9.84% of total genetic variation, respectively, with accessions forming six discernible clusters in PC1–PC2 space (Figure 4B). NJ phylogenetic analysis independently grouped all accessions into six distinct branches (Figure 4C). ADMIXTURE analysis produced a broadly concordant outcome (Figure 4D,E): cross-validation error declined continuously with increasing K, but the rate of decrease plateaued beyond K = 6. Integrating this pattern with the results from K-means clustering, PCA, and NJ phylogenetic analysis, all supporting six subgroups, K = 6 was adopted as the optimal solution, with subpopulations 1 through 6 containing 17, 74, 24, 97, 14, and 30 accessions (Table S4), respectively. This subpopulation assignment was incorporated into all subsequent GWAS models. The kinship heatmap showed elevated relatedness among accessions within subpopulations alongside clear genetic differentiation between them (Figure 4F), consistent with within-group homogenization and between-group divergence.

### 3.6. Genome-Wide Association Study of Salt Tolerance-Related Traits 

**Figure 5:**
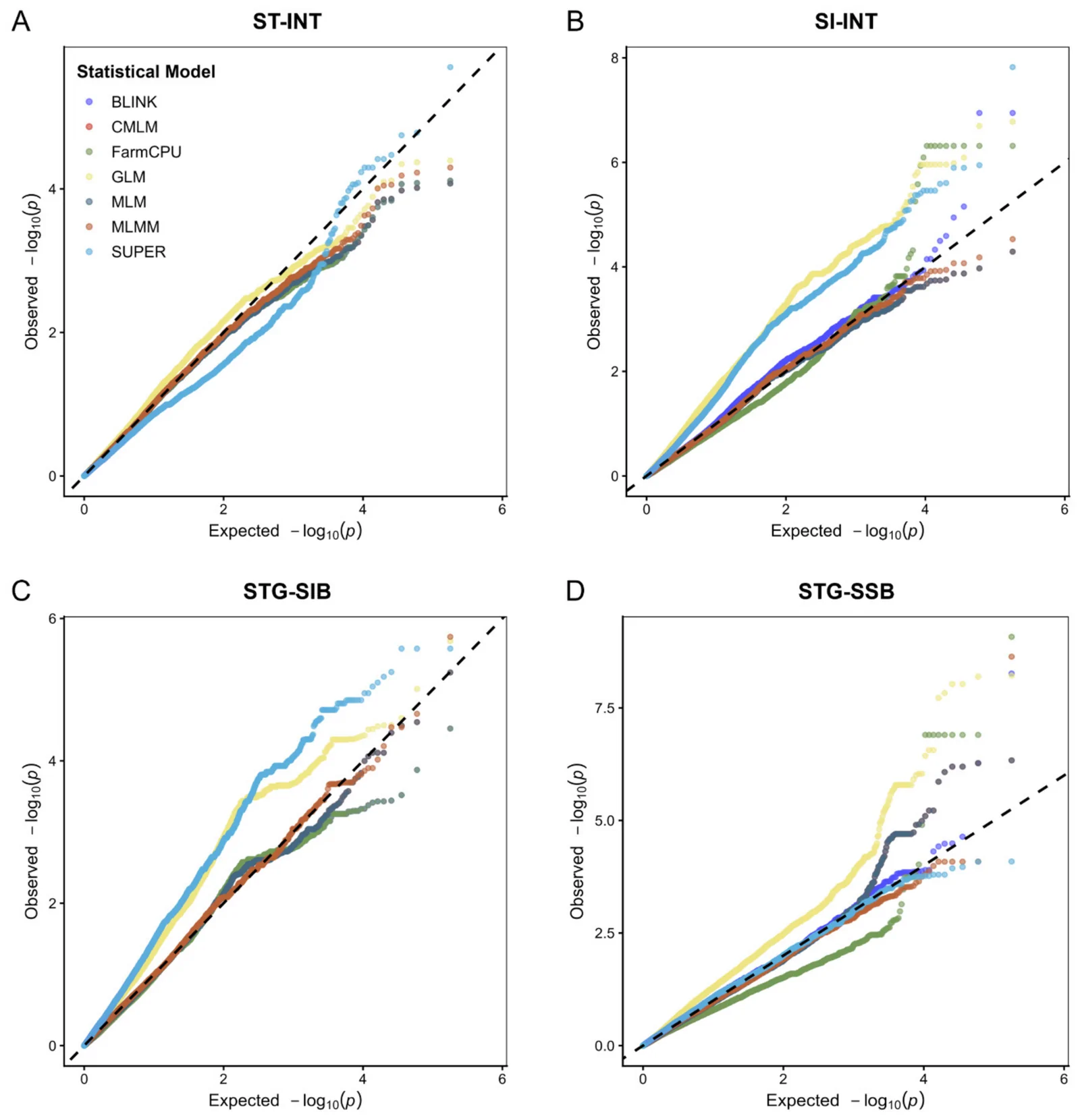
Quantile–quantile plots for ST-INT (**A**), SI-INT (**B**), STG-SIB (**C**), and STG-SSB (**D**) across the BLINK, CMLM, FarmCPU, GLM, MLM, MLMM, and SUPER models. ST-INT is the salt tolerance coefficient after inverse normal transformation at the emergence stage; SI-INT is the salt tolerance index after inverse normal transformation at the emergence stage; STG-SIB is the salt tolerance index grades after binary conversion at the emergence stage; STG-SSB is the salt tolerance grades following binary conversion at the seedling stage.

**Figure 6:**
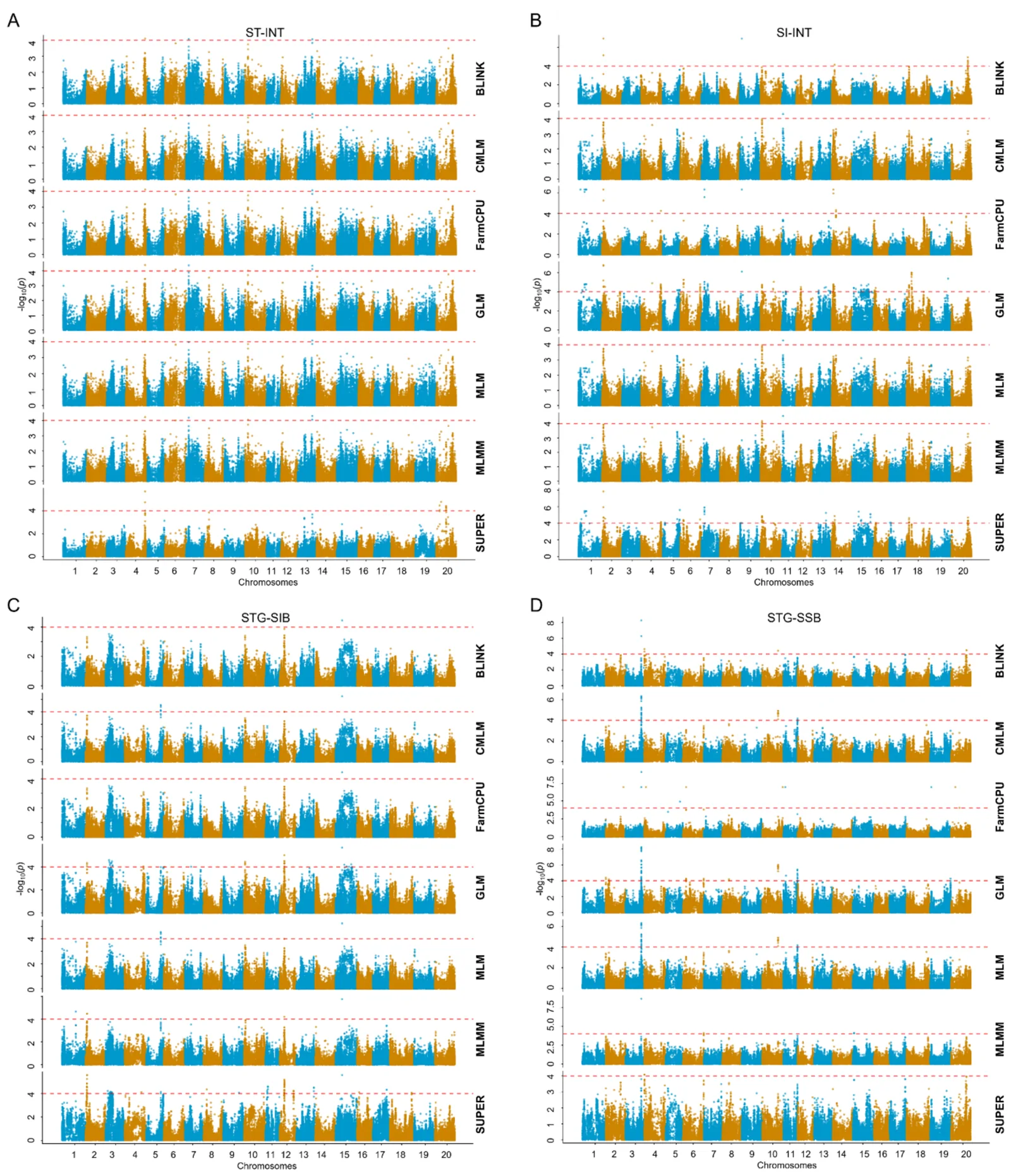
Manhattan plots for ST-INT (**A**), SI-INT (**B**), STG-SIB (**C**), and STG-SSB (**D**) across the BLINK, CMLM, FarmCPU, GLM, MLM, MLMM, and SUPER models. ST-INT is the salt tolerance coefficient after inverse normal transformation at the emergence stage; SI-INT is the salt tolerance index after inverse normal transformation at the emergence stage; STG-SIB is the salt tolerance index grades after binary conversion at the emergence stage; STG-SSB is the salt tolerance grades following binary conversion at the seedling stage.

GWAS was performed on the GAPIT platform for all four transformed traits, ST-INT, SI-INT, STG-SIB, and STG-SSB, using the full SNP dataset. To minimize false positives, only loci detected by at least three of the seven association models were retained. This yielded 60 significant SNPs (Table S5), which were consolidated into 19 QTLs based on LD decay distance (~50 kb), with the number of SNPs per QTL ranging from 1 to 20 (Table 2; Figure 5 and Figure 6).

Three QTLs for the emergence-stage trait ST-INT were mapped to chromosomes 4, 7, and 13 (Table 2). The strongest signal was qST-INT-04 (Gm04_46793849) on chromosome 4 (−log_10_(*P*) = 5.7). The chromosome 13 locus qST-INT-13 (Gm13_35700100) was situated approximately 0.87 Mb from *GmbZIP132* [46] and 1.39 Mb from *GmBIN2* [47] (Table S6). *GmbZIP132* encodes a bZIP transcription factor induced by salt and drought that increases salt tolerance at seed germination [46], whereas *GmBIN2* encodes a GSK3-type kinase also induced by both stresses and a positive regulator of stress tolerance [47].

Ten QTLs for the emergence-stage trait SI-INT were identified across chromosomes 1 (four loci), 2, 7, 9, 10, 11, and 14 (Table 2). The strongest signal came from qSI-INT-02 (Gm02_3640916–Gm02_3649811) on chromosome 2, spanning two SNPs. The chromosome 9 locus qSI-INT-09 (Gm09_5191652) mapped approximately 1.70 Mb from *GmPHD6* [48] (Table S6), whose overexpression in soybean hairy roots enhances salt tolerance while knockout increases sensitivity. The chromosome 10 locus qSI-INT-10 (Gm10_3521576–Gm10_3614887) lay approximately 2.54 Mb from *GmWRKY54* [49] and 2.14 Mb from *GmERF75* [50] (Table S6), both implicated in salt tolerance. The chromosome 14 locus qSI-INT-14 (Gm14_6728116) was located 0.76 Mb from a previously reported marker associated with *GsPRX*, a gene involved in oxidative stress responses [51] (Table S6). Each of these distances exceeds the LD decay distance estimated for this population (~50 kb), so the reported genes lie outside the linkage disequilibrium blocks of the associated loci and are not proposed here as causal.

Three QTLs for the emergence-stage binary trait STG-SIB were detected on chromosomes 5, 12, and 15 (Table 2), with qSTG-SIB-15 (Gm15_15831968) on chromosome 15 producing the strongest signal (−log_10_(*P*) = 5.74).

Three QTLs for the seedling-stage trait STG-SSB were mapped to chromosomes 3, 10, and 11 (Table 2). The chromosome 3 locus, qSTG-SSB-03 (Gm03_38525925–Gm03_38815291), was the most prominent, spanning a 0.29 Mb interval supported by 20 SNPs, with peak signal at Gm03_38688580 (−log_10_(*P*) = 9.08). This QTL lies approximately 192 kb from *Glyma03g32900* (*GmSALT3/GmCHX1*), the major seedling-stage salt tolerance gene cloned independently by Guan et al. [24] and Qi et al. [23] (Table S6). This distance exceeds the LD decay threshold (~50 kb), indicating that qSTG-SSB-03 and GmSALT3 likely reside in distinct LD blocks. qSTG-SSB-03 is therefore treated here as a locus separate from GmSALT3, and haplotype analysis across the interval would test whether the two signals are independent.

### 3.7. Candidate Gene Analysis

To refine candidate intervals and clarify local haplotype structure, candidate gene identification focused on qSI-INT-10 (Gm10_3521576–Gm10_3614887) for emergence-stage salt tolerance and qSTG-SSB-10 (Gm10_38386826–Gm10_38472859) for seedling-stage salt tolerance, the QTLs with the greatest number of significant SNPs outside the chromosome 3 locus. LDBlockShow analysis resolved seven LD blocks (Figure S5). For qSI-INT-10, a consistent LD block was detected with the GLM, MLMM, and SUPER models (Figure S5A–C), while for qSTG-SSB-10, the same block was identified in the BLINK, CMLM, GLM, and MLM models (Figure S5D–G).

From each stable LD block, the SNP with the highest −log_10_(*P*) value (i.e., the peak association SNP) was recorded as the peak association SNP, and protein-coding genes within the LD block boundaries extended by 50 kb on either side were taken as candidates, following the estimated LD decay distance. Within the emergence-stage QTL qSI-INT-10, 23 candidate genes were identified (Table 3), spanning functions including cell wall biosynthesis and remodeling (e.g., cellulose synthase A4 *Glyma.10G039600* and the KATAMARI1 homolog *Glyma.10G040700*, encoding a xyloglucan galactosyltransferase), transcriptional regulation (e.g., MYB-family transcription factor APL homolog *Glyma.10G039700* and auxin response factor 1 *Glyma.10G040400*), stress response and metabolism (e.g., glutathione S-transferase *Glyma.10G040000* and alcohol dehydrogenase 1 *Glyma.10G041000*), and protein kinase activity and methylation (e.g., *Glyma.10G041300* and *Glyma.10G040100*). For the seedling-stage QTL qSTG-SSB-10, 18 candidate genes were identified (Table 3), enriched for roles in DNA metabolism and repair (e.g., RecQ family helicase *Glyma.10G148300*, Werner syndrome-like exonuclease *Glyma.10G148600*, and single-stranded DNA-binding protein *Glyma.10G149700*) and stress response (e.g., drought-induced protein 19 *Glyma.10G149200* and calmodulin-binding protein *Glyma.10G148700*), along with four uncharacterized proteins, two proteins annotated as unknown, and one protein of unknown function. Based on functional annotation and established relevance to salt stress, four genes, namely *Glyma.10G040000*, *Glyma.10G148700*, *Glyma.10G149200*, and *Glyma.10G149600*, were prioritized as putative candidate genes and are discussed below.

## 4. Discussion

Salt tolerance in soybean is developmentally stage-specific [52], and tolerance at germination often exceeds that at emergence. Timely post-sowing irrigation can reduce surface salinity and partially mitigate this vulnerability [53]. In soils with dry-weight salt content below 1.0%, emergence is reduced even when germination rates remain high across genotypes. This distinction is illustrated by the Williams cultivar, which maintained a germination rate of 81% at 330 mM NaCl, yet seedling growth fell to 5% under the lower concentration of 220 mM NaCl [54]. Under field conditions, seeds may germinate successfully in saline soil but fail to penetrate a salt-hardened surface crust. This underscores the need to evaluate salt tolerance, map relevant loci, and identify candidate genes specifically at the emergence stage.

Relatively few soybean salt-tolerance genes have been cloned through forward genetics to date, including the seedling-stage genes *GmSALT3/GmCHX1* [23,24] and *GsERD15B* [55], and the germination-stage gene *GmCDF1* [56]. Our GWAS yielded four putative candidate genes, one emergence-stage and three seedling-stage. The emergence-stage candidate *Glyma.10G040000*, located within qSI-INT-10, encodes a GST. GSTs are well-established contributors to plant salt tolerance, acting through ROS homeostasis and thereby limiting salt-induced oxidative damage; transgenic overexpression of GST genes increases salt tolerance [57]. MYB and WRKY transcription factors act upstream of GST expression, placing these enzymes within broader stress-response networks [58]. In soybean specifically, the tau-class member GmGSTU23 enhances salt tolerance through elevated glutathione transferase activity [59], suggesting that *Glyma.10G040000* may increase tolerance through comparable biochemical activity in ROS detoxification. However, eFP Browser analysis showed low expression in roots and root hairs under normal conditions, warranting further investigation of its expression under salt stress.

The three seedling-stage putative candidate genes all map to qSTG-SSB-10. *Glyma.10G148700* encodes a calmodulin-binding protein, which decodes salt-induced cytosolic Ca^2+^ transients and translates them into adaptive physiological responses. In rice, the calmodulin-binding protein OsMSR2 is induced by salt stress, and its overexpression in *Arabidopsis* increases tolerance through ABA signaling and downstream stress-response gene expression [60]. Conversely, the *Medicago truncatula* ortholog MtCML40 is a negative regulator: its overexpression increases salt sensitivity, accompanied by Na^+^ accumulation in shoots and suppression of the sodium efflux genes *MtHKT1;1* and *MtHKT1;2* [61]. The contrasting roles of these orthologs show that calmodulin-binding proteins act in the salt-stress response with species-dependent direction, which leaves the direction of any effect of *Glyma.10G148700* open to testing. However, its expression in roots was low under normal conditions.

*Glyma.10G149200* encodes drought-induced protein 19 (Di19), with documented roles in plant salt tolerance. In soybean, GmDi19-5 is a negative regulator that interacts with the E3 ubiquitin ligase GmPUB21 and is degraded in an ABA-dependent manner, enabling fine-tuned modulation of stress responses [62]. In maize, ZmDi19-1 enhances salt tolerance by activating downstream stress-responsive genes and increasing antioxidant capacity [63], while in cotton, GhDi19-3 and GhDi19-4 improve salt tolerance through Ca^2+^ and ABA signaling and concurrent ROS scavenging [64]. Di19 proteins therefore function where several stress pathways converge, which supports *Glyma.10G149200* as a candidate for salt tolerance. Moderate basal expression in roots is consistent with a potential role in stress signal transduction.

*Glyma.10G149600* encodes a PP2C family protein. In *Arabidopsis*, PP2Cs are negative regulators of ABA signaling and can directly interact with and inhibit the plasma membrane Na^+^/H^+^ antiporter SOS1, a sodium efflux transporter [65]. In peanut, PP2C genes including *AhPP2C45* and *AhPP2C134* are upregulated under salt stress [66]. Through direct action on sodium transport and compartmentalization, PP2Cs function in adaptation to saline environments, which supports a role for *Glyma.10G149600* in soybean salt tolerance. Expression peaked in root tips, a site of salt perception, and increased following rhizobium inoculation at 12 and 48 h, consistent with the stress-responsive expression pattern reported for PP2C genes in other species.

These candidate genes await experimental validation, and their roles in soybean salt tolerance should be tested through salt-stress expression profiling and functional assays via transgenic or gene-editing approaches. Moreover, the QTLs reported here were identified under controlled NaCl conditions and may not fully reflect the genetic architecture of tolerance to complex saline–alkaline soils in the field. Field transferability of these loci warrants validation through multi-environment trials prior to marker-assisted breeding deployment.

Beyond gene discovery, translating genomic findings into practical breeding requires broader consideration of how and when salt stress is evaluated. Unlike many abiotic stresses, salt-alkali stress persists throughout most or all of the crop growth cycle [67]. Current soybean salt-tolerance research is often focused on seedlings, assuming that early vegetative vigor reliably predicts agronomic performance. This assumption does not always hold, as excessive salinity can redirect photosynthate from growth to stress tolerance. Indeed, some salt-tolerant genotypes show early vigor comparable to sensitive genotypes yet outperform them under field conditions [53]. Multi-stage field evaluation therefore carries information that single-stage assays do not. Furthermore, most laboratory assays use NaCl as the sole treatment, while salinized soils worldwide vary widely in pH and ionic composition [68]. Salinization and alkalization frequently co-occur, yet their combined effects on plant growth are not additive. The severity ranking from greatest to least is saline–alkaline stress, alkaline stress, then salt stress alone, and mixed saline–alkaline injury substantially exceeds the sum of the individual stresses [69,70]. As research on mixed saline–alkaline tolerance remains limited, future efforts should extend beyond single-salt or single-alkali treatments to encompass the complex stress combinations encountered in the field.

## 5. Conclusions

GWAS combining ZDX1 SNP array data from 256 soybean accessions with salt-tolerance phenotypes at emergence and seedling stages identified 60 significant SNPs consolidated into 19 QTLs, with 16 associated with emergence-stage and 3 with seedling-stage salt tolerance. Previously reported stress-tolerance genes reside within the broader chromosomal regions of 5 of these QTLs; the seedling-stage QTL qSTG-SSB-03 lies 192 kb from the major salt-tolerance gene *GmSALT3*. This distance is beyond the linkage disequilibrium decay of this population. Four putative candidate genes were prioritized: the emergence-stage gene *Glyma.10G040000*, encoding a glutathione S-transferase, and the seedling-stage genes *Glyma.10G148700*, *Glyma.10G149200*, and *Glyma.10G149600*, encoding a calmodulin-binding protein, drought-induced protein 19, and a PP2C family protein, respectively. These candidates await experimental validation through gene expression analysis and functional assays.

## Figures and Tables

**Table 1:** Descriptive statistics of salt tolerance at emergence and seedling stages.

Traits	Maximum	Minimum	Mean	Variance	*SD* ^a^	Skewness	Kurtosis
ST ^b^	100.00	33.33	89.32	128.82	11.35	−1.90	4.60
SI ^c^	1.00	0.09	0.62	0.05	0.23	−0.50	−0.66
STG-SI ^d^	5.00	1.00	2.43	1.30	1.14	0.52	−0.63
STG-SS ^e^	5.00	1.00	2.30	1.54	1.24	0.66	−0.63
ST-INT ^f^	1.34	−2.66	−0.02	0.88	0.94	−0.26	−0.57
SI-INT ^g^	2.66	−2.42	0	0.96	0.98	0	−0.28
STG-SIB ^h^	1.00	0	0.60	0.24	0.49	−0.41	−1.84
STG-SSB ^i^	1.00	0	0.63	0.24	0.49	−0.51	−1.74

^a^ *SD* is standard deviation; ^b^ ST is salt tolerance coefficient at emergence stage; ^c^ SI is salt tolerance index at emergence stage; ^d^ STG-SI is salt tolerance index grades at emergence stage; ^e^ STG-SS is salt tolerance grades at seedling stage; ^f^ ST-INT is salt tolerance coefficient after inverse normal transformation at emergence stage; ^g^ SI-INT is salt tolerance index after inverse normal transformation at emergence stage; ^h^ STG-SIB is salt tolerance index grades after binary conversion at emergence stage; ^i^ STG-SSB is salt tolerance grades following binary conversion at seedling stage.

**Table 2:** Quantitative trait loci (QTL) for salt tolerance identified by GWAS in this study.

QTL Name	Associated Traits	Chr.	SNP Interval	SNP Number Contained	Highest Association SNP	−log_10_(*P*)
qST-INT-04	ST-INT ^a^	4	Gm04_46793849	1	Gm04_46793849	5.70
qST-INT-07	ST-INT	7	Gm07_6279424	1	Gm07_6279424	4.37
qST-INT-13	ST-INT	13	Gm13_35700100	1	Gm13_35700100	4.35
qSI-INT-01-1	SI-INT ^b^	1	Gm01_14874637-14927241	2	Gm01_14874637	6.09–6.32
qSI-INT-01-2	SI-INT	1	Gm01_17436477	1	Gm01_17436477	6.32
qSI-INT-01-3	SI-INT	1	Gm01_18506780	1	Gm01_18506780	6.32
qSI-INT-01-4	SI-INT	1	Gm01_19151980	1	Gm01_19151980	6.32
qSI-INT-02	SI-INT	2	Gm02_3640916-3649811	2	Gm02_3649811	6.78–7.82
qSI-INT-07	SI-INT	7	Gm07_7238076-7250804	2	Gm07_7238076	5.90–6.32
qSI-INT-09	SI-INT	9	Gm09_5191652	1	Gm09_5191652	6.95
qSI-INT-10	SI-INT	10	Gm10_3521576-3614887	3	Gm10_3521576, Gm10_3614887	4.73–4.84
qSI-INT-11	SI-INT	11	Gm11_2844635	1	Gm11_2844635	4.53
qSI-INT-14	SI-INT	14	Gm14_6728116	1	Gm14_6728116	4.86
qSTG-SIB-05	STG-SIB ^c^	5	Gm05_35273972-35303385	2	Gm05_35303385	4.39–4.50
qSTG-SIB-12	STG-SIB	12	Gm12_11112335	1	Gm12_11112335	5.18
qSTG-SIB-15	STG-SIB	15	Gm15_15831968	1	Gm15_15831968	5.74
qSTG-SSB-03	STG-SSB ^d^	3	Gm03_38525925-38815291	20	Gm03_38688580	4.41–9.08
qSTG-SSB-10	STG-SSB	10	Gm10_38386826-38472859	16	Gm10_38398774	5.20–6.01
qSTG-SSB-11	STG-SSB	11	Gm11_34520086-34522630	2	Gm11_34522630	4.67–4.81

^a^ ST-INT is the salt tolerance coefficient after inverse normal transformation at the emergence stage; ^b^ SI-INT is the salt tolerance index after inverse normal transformation at the emergence stage; ^c^ STG-SIB is the salt tolerance index grades after binary conversion at the emergence stage; ^d^ STG-SSB is the salt tolerance grades after binary conversion at the seedling stage.

**Table 3:** Candidate genes and functional annotations.

QTL Name	Gene ID	Chr.	Start	End	Gene Function
qSI-INT-10	*Glyma.10G039200*	Gm10	3476029	3482210	Tetratricopeptide repeat protein 7B-like
	*Glyma.10G039300*	Gm10	3484313	3487965	Rho GDP-dissociation inhibitor 1
	*Glyma.10G039400*	Gm10	3489552	3494424	Exocyst complex component EXO84B-like
	*Glyma.10G039500*	Gm10	3495933	3498821	Uncharacterized protein LOC100793067
	*Glyma.10G039600*	Gm10	3498156	3501931	Cellulose synthase A4
	*Glyma.10G039700*	Gm10	3509365	3515838	Myb family transcription factor APL-like
	*Glyma.10G039800*	Gm10	3523133	3524397	Unknown protein
	*Glyma.10G039900*	Gm10	3528125	3529563	60S ribosomal L23-like protein
	*Glyma.10G040000*	Gm10	3532303	3534011	Glutathione S-transferase family protein
	*Glyma.10G040100*	Gm10	3534381	3541727	Histone-lysine N-methyltransferase SUVR2-like
	*Glyma.10G040200*	Gm10	3547051	3547805	Mitochondrial import receptor subunit TOM9-2-like
	*Glyma.10G040300*	Gm10	3548744	3550403	Uncharacterized protein LOC102667717
	*Glyma.10G040400*	Gm10	3557161	3561101	Auxin response factor 1
	*Glyma.10G040500*	Gm10	3561542	3565454	ATP-binding/protein serine/threonine kinase
	*Glyma.10G040600*	Gm10	3568550	3571109	Photosystem II reaction center PSB28 protein
	*Glyma.10G040700*	Gm10	3572769	3575692	Xyloglucan galactosyltransferase KATAMARI1-like
	*Glyma.10G040800*	Gm10	3585796	3589311	Proline-rich protein precursor
	*Glyma.10G040900*	Gm10	3591102	3596087	Tetratricopeptide repeat protein 4 homolog
	*Glyma.10G041000*	Gm10	3597307	3601274	Alcohol dehydrogenase 1
	*Glyma.10G041100*	Gm10	3601867	3606814	3-oxoacyl-[acyl-carrier-protein] synthase
	*Glyma.10G041200*	Gm10	3613943	3614897	RmlC-like cupins superfamily protein
	*Glyma.10G041300*	Gm10	3621767	3629177	Protein kinase superfamily protein
	*Glyma.10G041400*	Gm10	3654431	3661105	PfkB-like carbohydrate kinase family protein
qSTG-SSB-10	*Glyma.10G148100*	Gm10	38339322	38341729	Uncharacterized protein DDB_G0271670-like
	*Glyma.10G148200*	Gm10	38353363	38357916	Uncharacterized protein LOC100777900
	*Glyma.10G148300*	Gm10	38372070	38378539	RecQ family ATP-dependent DNA helicase
	*Glyma.10G148400*	Gm10	38379206	38380060	Unknown protein
	*Glyma.10G148500*	Gm10	38381421	38382250	Protein of unknown function (DUF3511)
	*Glyma.10G148600*	Gm10	38382588	38389222	Werner Syndrome-like exonuclease-like
	*Glyma.10G148700*	Gm10	38415381	38418722	Calmodulin-binding protein
	*Glyma.10G148800*	Gm10	38420217	38425350	Importin subunit alpha-1b
	*Glyma.10G148900*	Gm10	38427372	38430979	DNA polymerase III, epsilon subunit-like protein
	*Glyma.10G149000*	Gm10	38431647	38436740	Syntaxin of plants 43
	*Glyma.10G149100*	Gm10	38441580	38441963	Unknown protein
	*Glyma.10G149200*	Gm10	38464899	38467826	Drought-induced 19
	*Glyma.10G149300*	Gm10	38468901	38469373	Uncharacterized protein LOC100797448
	*Glyma.10G149400*	Gm10	38469764	38473037	PHD and RING finger domain-containing protein 1
	*Glyma.10G149500*	Gm10	38493040	38499888	ATP-binding microtubule motor family protein
	*Glyma.10G149600*	Gm10	38511278	38514578	Protein phosphatase 2C family protein
	*Glyma.10G149700*	Gm10	38516493	38521186	Single-stranded DNA-binding protein
	*Glyma.10G149800*	Gm10	38520715	38521086	Uncharacterized protein LOC102667166

## Data Availability

The original data presented in the study are openly available at Supplementary Materials.

## Supplementary Materials

The following supporting information can be downloaded at: https://www.mdpi.com/article/10.3390/biology15161409/s1, Figure S1: Representative phenotypes of each salt tolerance grade at the emergence stage; Figure S2: Representative phenotypes of salt-injury symptoms at the seedling stage; Figure S3: Analysis of variance of soybean emergence rate for salt treatment and control; Figure S4: Phenotypic distributions of salt tolerance traits. Frequency histograms for ST-INT (A) and SI-INT (B); bar charts for STG-SIB (C) and STG-SSB (D); Figure S5: Candidate regions for qSI-INT-10 (SI-INT) in the GLM, MLMM, and SUPER models (A–C) and for qSTG-SSB-10 (STG-SSB) in the GLM, MLM, CMLM, and BLINK models (D–G); Table S1: Detailed information for 256 soybean accessions; Table S2: Salt damage symptoms and salt tolerance index grades at soybean emergence stage under salt stress; Table S3: Grading criteria and corresponding salt-injury symptoms at the soybean seedling stage under salt stress; Table S4: Accessions were assigned to six subpopulations (Pop 1–6) based on ADMIXTURE analysis at K = 6; Table S5: SNPs significantly associated with salt-tolerance-related traits; Table S6: Previously reported stress-tolerance genes located near QTLs identified in this study.
