# Supplementary material for: Genome-Wide Association Identifies Candidate Genes for Salt Tolerance in Soybean at Emergence and Seedling Stages
Source: Biology (Basel). 2026 Aug 17;15(16):1409. doi: 10.3390/biology15161409 (PMC13509925; doi:10.3390/biology15161409)

Figure captions

Figure S1. Representative phenotypes of each salt tolerance grade at the emergence stage.

Figure S2. Representative phenotypes of salt-injury symptoms at the seedling stage (from Liu [42]).

Figure S3. Analysis of variance of soybean emergence rate for salt treatment and control. ***, significant level under 0.001 for Pearson correlation test.

Figure S4. Phenotypic distributions of salt tolerance traits. Frequency histograms for ST-INT (**A**) and SI-INT (**B**); bar charts for STG-SIB (**C**) and STG-SSB (**D**). ST-INT is salt tolerance coefficient after inverse normal transformation at emergence stage; SI-INT is salt tolerance index after inverse normal transformation at emergence stage; STG-SIB is salt tolerance index grades after binary conversion at emergence stage; STG-SSB is salt tolerance grades following binary conversion at seedling stage.

Figure S5. Candidate regions for qSI-INT-10 (SI-INT) in the GLM, MLMM, and SUPER models (**A**–**C**) and for qSTG-SSB-10 (STG-SSB) in the GLM, MLM, CMLM, and BLINK models (**D**–**G**).

Figure S1. Representative phenotypes of each salt tolerance grade at the emergence stage.

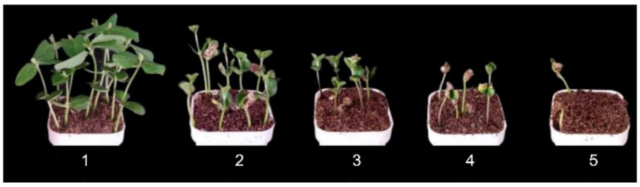

Figure S2. Representative phenotypes of salt-injury symptoms at the seedling stage (from Liu [42]).

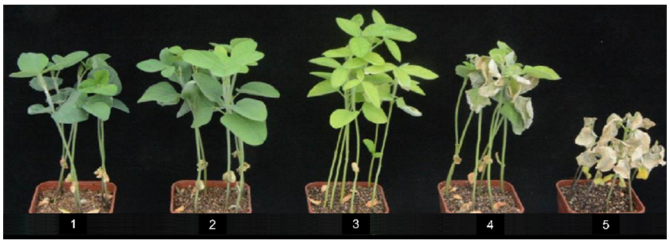

Figure S3. Analysis of variance of soybean emergence rate for salt treatment and control. ***, significant level under 0.001 for Pearson correlation test.

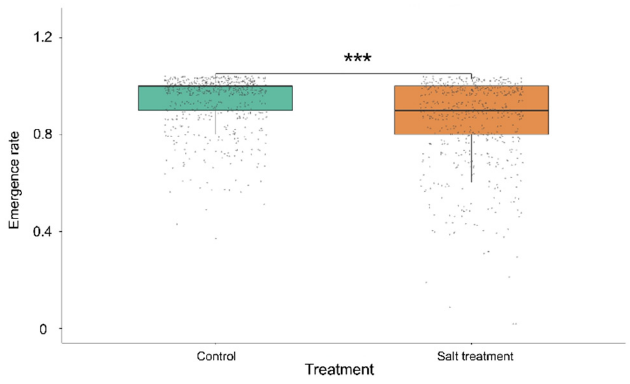

Figure S4. Phenotypic distributions of salt tolerance traits. Frequency histograms for ST-INT (**A**) and SI-INT (**B**); bar charts for STG-SIB (**C**) and STG-SSB (**D**). ST-INT is salt tolerance coefficient after inverse normal transformation at emergence stage; SI-INT is salt tolerance index after inverse normal transformation at emergence stage; STG-SIB is salt tolerance index grades after binary conversion at emergence stage; STG-SSB is salt tolerance grades following binary conversion at seedling stage.

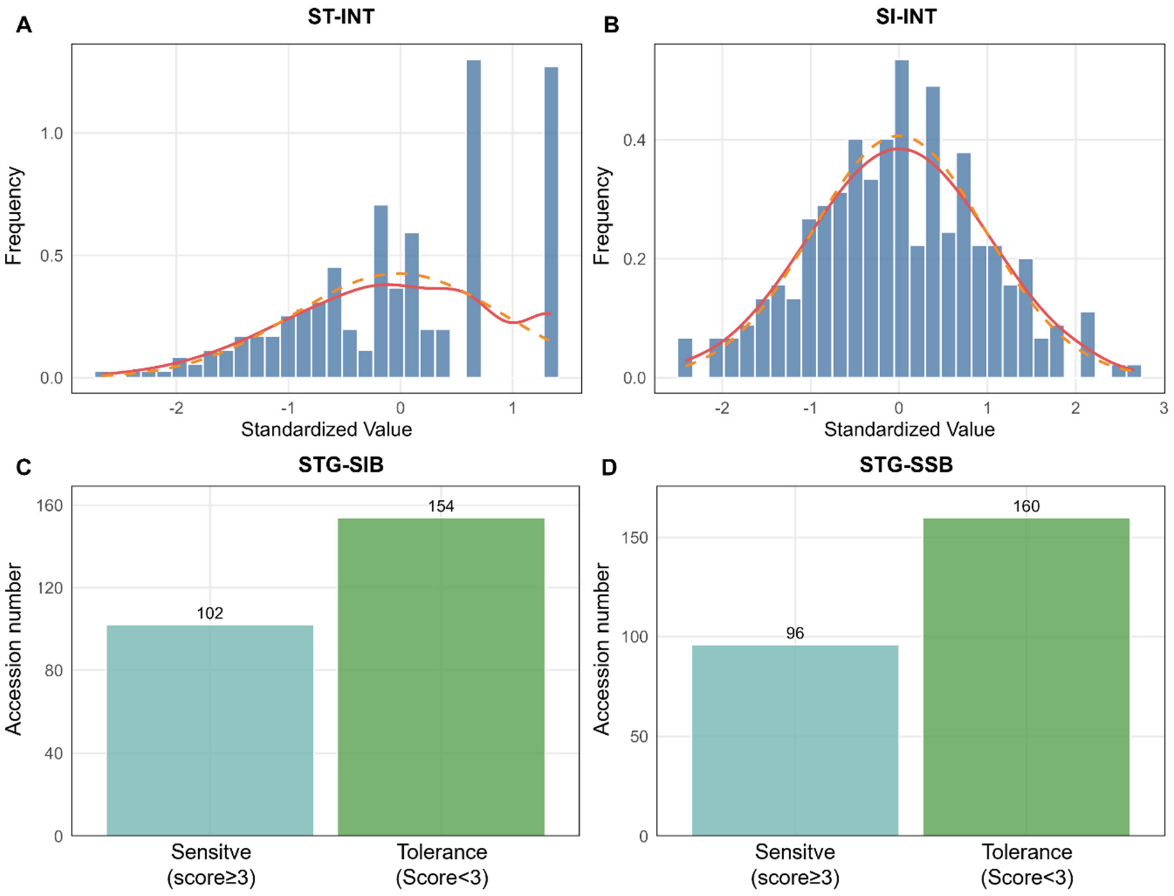

Figure S5. Candidate regions for qSI-INT-10 (SI-INT) in the GLM, MLMM, and SUPER models (**A**–**C**) and for qSTG-SSB-10 (STG-SSB) in the GLM, MLM, CMLM, and BLINK models (**D**–**G**).

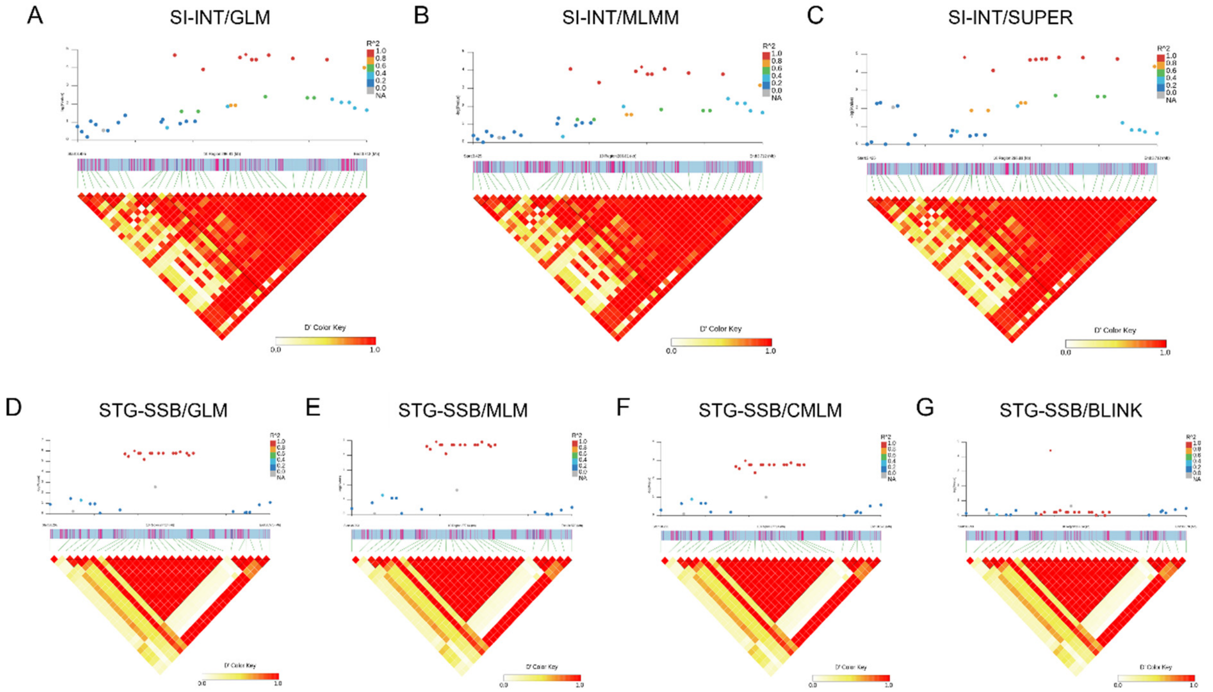

Supplement: Supplementary file 1 [file biology-15-01409-s001.zip › Supplemental Figs and Tables/Figures.pdf]
