# Supplementary material for: Genome-Wide Association Identifies Candidate Genes for Salt Tolerance in Soybean at Emergence and Seedling Stages
Source: Biology (Basel). 2026 Aug 17;15(16):1409. doi: 10.3390/biology15161409 (PMC13509925; doi:10.3390/biology15161409)
Supplement: Supplementary file 1 [file biology-15-01409-s001.zip › Supplemental Figs and Tables/Tables.pdf]

Table S1. Detailed information for 256 soybean accessions.

No.	Test ID	Name
1	208140740105_R01C02	2170409
2	205730040054_R01C01	2170425
3	208140740105_R04C02	2170427
4	205740000164_R10C02	2170432
5	208140740006_R09C02	2170436
6	208140740105_R05C02	2170469
7	208140740086_R02C01	2170471
8	208141520015_R11C01	2170475
9	208140740077_R10C01	2170477
10	208140740077_R08C01	2261363
11	208141520012_R09C01	2261372
12	208140740077_R07C02	2261453
13	208140740077_R01C02	2270208
14	208140740006_R03C02	2270961
15	208141520012_R11C02	2271072
16	208140740086_R11C01	2271360
17	208140740077_R07C01	2271387
18	208140740077_R04C02	2271465
19	208141520015_R05C02	2271510
20	208141520015_R03C02	2271516
21	208141520012_R01C01	2271627
22	208140740086_R10C01	2271693
23	208141520012_R04C02	2271714
24	208140740108_R01C02	20P5
25	208141520015_R11C02	20SQ041
26	208140740077_R10C02	20SQ301
27	208141520012_R03C02	21MD5056-060
28	208141520015_R02C01	21MD7043
29	208140740105_R03C01	21MD8019-021
30	205740000001_R05C01	21P009-012
31	205740000001_R07C02	21P029-032
32	205740000001_R08C02	21P037-040
33	208140740105_R06C02	21P041-044
34	205740000001_R09C02	21P049-052
35	205740000001_R10C02	21P061-064
36	208140740077_R02C01	21P073-076
37	205740000001_R11C02	21P081-084
38	205740000001_R12C02	21P085-088
39	208140740105_R02C01	21P093-096
40	208140740006_R12C02	21SQP025-028
41	208141520012_R03C01	21SQP049-052
42	208140740086_R09C02	21SQJian0004-0006
43	208140740010_R03C02	21Jian0004
44	208141520012_R08C01	21Jian0005

45	208140740086_R07C01	21Jian0009
46	208140740006_R10C01	21Jian0014-0015
47	208140740105_R10C01	21Jian0017
48	205740000164_R09C02	21Jian0019
49	208141520015_R10C01	21Jian0031-0033
50	208141520012_R05C01	21Jian0034-0036
51	208140740006_R01C01	21Jian0040-0042
52	208140740006_R12C01	21Jian0043-0045
53	205740000001_R03C02	21Jian0058-0060
54	208140740086_R12C02	21Jian0064-0066
55	205740000001_R04C02	21Jian0094-0096
56	208140740006_R06C02	21Jian0103-0105
57	205740000001_R05C02	21Jian0151-0153
58	205740000001_R06C02	21Jian0172-0174
59	205740000001_R11C01	21Jian0175-0177
60	208140740006_R07C02	21Jian0181-0183
61	208141520015_R09C01	21Jian0184-0186
62	208140740086_R03C02	21Jian0196-0198
63	208140740077_R11C01	21Jian0211-0213
64	208140740105_R11C01	21Jian0214-0216
65	205740000001_R02C02	21Jian0229-0231
66	205740000001_R09C01	21Jian0253-0255
67	208140740006_R11C02	21Jian0262-0264
68	208140740105_R02C02	21Jian0268-0270
69	208140740105_R06C01	21Jian0277-0278
70	208140740108_R01C01	21Jian0447-0449
71	205740000001_R10C01	21Jian0509-0511
72	208141520012_R11C01	21Jian0518-0520
73	205740000001_R03C01	21Jian0524-0526
74	205740000164_R08C02	21Jian0533-0535
75	208140740105_R12C01	21Jian0566-0568
76	205740000164_R07C02	21Jian0593-0595
77	205740000164_R06C02	21Jian0596-0598
78	208140740010_R04C02	21Jian0704-0706
79	208140740105_R03C02	21Jian0779-0781
80	208141520015_R08C02	21Jian0788-0790
81	208140740086_R06C02	21Jian0800-0802
82	208140740077_R09C01	21Jian0806-0808
83	208140740086_R09C01	21Jian0812-0814
84	205740000164_R04C02	21Jian0821-0823
85	208141520015_R03C01	21Jian0848-0850
86	208141520015_R04C02	21Jian0920-0922
87	208140740086_R05C02	21Jian0941-0943
88	208140740077_R03C01	21Jian0944-0946
89	208141520015_R06C01	21Jian1007-1009
90	208140740086_R11C02	21Jian1016-1018

91	208140740077_R11C02	21Jian1019-1021
92	208140740077_R01C01	21Jian1070-1072
93	208141520015_R05C01	21Jian1109-1111
94	208140740105_R07C02	21Jian1148-1150
95	205740000001_R07C01	21Jian1169-1171
96	205730040054_R02C01	21Jian1178-1180
97	208140740006_R06C01	21Jian1184-1186
98	208140740105_R12C02	21Jian1187-1189
99	208140740105_R11C02	21Jian1193-1195
100	205740000001_R08C01	21Jian1196-1198
101	208140740105_R04C01	21Jian1205-1207
102	208140740006_R05C01	21Jian1231
103	205730040054_R05C01	21Jian1246
104	208140740077_R05C01	21Jian1249
105	208141520015_R12C02	21Jian1250
106	208140740006_R08C01	21Jian1306
107	208140740086_R05C01	21Jian1307
108	208141520012_R06C01	21Jian1348
109	208140740006_R07C01	21Jian1349
110	208140740077_R08C02	21Jian1352
111	208141520012_R06C02	21Jian1361
112	208141520012_R12C02	21Jian1384
113	208140740077_R12C01	21Jian1386
114	208140740086_R02C02	21Jian1398
115	208140740077_R12C02	21Jian1404
116	208140740105_R07C01	21Jian1405
117	208141520012_R07C02	21Jian1407
118	208141520015_R10C02	21Jian1409
119	208140740086_R01C02	21Jian1414
120	208140740105_R10C02	21Jian1424
121	208141520012_R02C02	2280037-0039
122	208140740077_R03C02	22P333
123	208140740086_R03C01	22XX6253
124	208141520012_R01C02	22XX70040
125	208140740006_R08C02	22XX8001
126	208140740077_R06C02	22XXMS2260
127	208141520012_R05C02	22XXNMD10025
128	208140740045_R02C01	22XXNMD10031
129	208141520015_R08C01	22XXNMD6082
130	208140740086_R04C02	22XXNMD6127
131	208140740006_R02C01	22XXNMD6130
132	208140740105_R01C01	22XXNMD6139
133	208141520015_R01C01	22XXNMD6145
134	208140740006_R04C01	22XXNMD6148
135	208140740006_R10C02	22XXNMD7103
136	208141520012_R02C01	22XXSQJian058

137	208140740086_R10C02	22XXSQJian178
138	208140740086_R01C01	22XXJian1091
139	208141520015_R02C02	22XXJian1109
140	208140740006_R04C02	22XXJian1112
141	208140740086_R08C02	22XXJian1268
142	208140740077_R05C02	22XXJian1298
143	208140740105_R05C01	22XXJian1310
144	208141520015_R07C01	22XXJian1322
145	208140740006_R09C01	22XXJian1337
146	208141520012_R08C02	22XXJian1376
147	208141520012_R10C01	22Jian0125
148	208140740045_R01C01	22Jian1196
149	208141520012_R12C01	22Jian1223
150	208141520012_R04C01	22Jian1400
151	208141520015_R04C01	22Jian1409
152	208141520012_R10C02	22Jian1412
153	208140740086_R07C02	22Jian1418
154	208140740086_R12C01	22Jian1697
155	208141520012_R09C02	22Jian1730
156	208140740006_R02C02	22Jian3580
157	208270070074_R07C01	23J2077-2080
158	208270070074_R08C01	23J3205-3207
159	208270070074_R11C01	23J3271-3273
160	208270070074_R09C01	23J3391-3393
161	208270070045_R10C02	23J3415-3417
162	208270070045_R08C02	23J3466-3468
163	208270070074_R01C01	23J3691-3693
164	208270070045_R05C02	23J3811-3813
165	208270070074_R02C01	23J3835-3837
166	208270070074_R05C01	23J3841-3843
167	208270070045_R11C01	23J3898-3900
168	208270070045_R04C02	23J3904-3906
169	208270070074_R11C02	23J5625-5628
170	208270070045_R10C01	23J5649-5652
171	208270070045_R11C02	23J5945-5948
172	208270070074_R01C02	23J5953-5956
173	208270070045_R01C02	23J6004-6006
174	208270070074_R10C02	23J6328-6330
175	208270070045_R12C01	23J6538-6540
176	208270070074_R06C02	23J6541-6543
177	208270070045_R07C02	23J6547-6549
178	208270070045_R09C02	23J6565-6567
179	208270070074_R10C01	23J6574-6576
180	208270070074_R05C02	23J6601-6603
181	208270070074_R08C02	23J6604-6606
182	208270070074_R09C02	23J6820-6822

183	208270070074_R12C02	23J6850-6851
184	208270070074_R03C02	23J6888
185	208270070074_R07C02	23J7001-7004
186	208270070074_R12C01	23J7069-7072
187	208270070074_R04C02	23J7465-7468
188	208270070045_R02C02	23J7921-7924
189	208270070045_R06C02	23J7929-7932
190	208270070074_R03C01	23MD049-051
191	208270070074_R02C02	23MD061-063
192	208270070074_R04C01	23MD121-123
193	208270070045_R12C02	23MD175-177
194	208270070045_R03C02	23MD724-726
195	208270070074_R06C01	23MD730-732
196	208140740010_R10C01	782-1
197	208140740045_R03C01	860-6
198	208140740010_R05C01	a+a'Yaji
199	208140740108_R04C02	aYaji
200	208140740059_R08C02	PI 416937
201	208140740045_R09C01	Cangdou 15
202	203568950003_R01C01	Fendou 78
203	208140740105_R09C02	Fendou 95
204	208140740010_R03C01	Gong552
205	205740000133_R08C01	Handou 13
206	205740000133_R09C01	Handou 14
207	208140740108_R02C01	Huangdou 12
208	208140740108_R02C02	Jinong 58
209	208140740059_R09C02	Jiyu 397
210	208140740108_R10C02	Jiyu 548
211	208140740010_R11C01	JiHJ116
212	203757450074_R02C01	Jidou 12
213	208140740045_R04C02	Jinhuang 10
214	205740000133_R02C02	Jinke 5
215	203757450034_R05C01	Jinyi 51
216	208140740108_R07C01	Kedou 35
217	208141520015_R09C02	Kefeng 29
218	205595660042_R11C02	Longzhonghuang 601
219	208140740010_R02C02	Mengkedou 12
220	208140740010_R01C01	NinghuangLD89
221	205740000133_R04C01	Ningjingdou 7
222	203568950003_R04C01	Qihuang 34
223	208140740086_R04C01	Qihuang 42
224	205600730158_R08C01	Shanning 17
225	205740000133_R07C02	Shanning 21
226	208140740108_R12C01	Shanning 27
227	208140740045_R08C02	Shanning 29
228	203568950003_R10C01	Shengdou 10

229	208140740108_R11C01	Shengdou 24
230	205740000133_R12C01	Shi 885
231	203757450034_R12C01	Shidou 17
232	203757450122_R03C02	Sudou 13
233	205739190117_R06C01	Suike 23
234	205730040062_R11C01	Wandou 37
235	203757450075_R07C01	Weidou 1897
236	203550230076_R02C02	Wuxing 4
237	208140740105_R08C01	Xiangfeng 2
238	203757450075_R02C02	Xiangfeng 4
239	203757420057_R04C01	Xingdou 5
240	208141520012_R07C01	Xu9416-6bai
241	205739190011_R12C01	Xudou 14
242	203581760004_R10C02	Xudou 18
243	203581760004_R12C02	Xudou 20
244	205739190106_R04C01	Xudou 21
245	205740000074_R12C01	Xudou 22
246	205740000022_R11C02	Xudou 23
247	205740000074_R01C02	Xudou 24
248	205730040028_R09C02	Xudou 25
249	205600730192_R07C01	Xudou 9
250	208140740108_R11C02	Yundou 202
251	203757450120_R01C01	Yundou 1
252	203757450074_R07C02	Zheng 1311
253	208140740077_R04C01	Zheng 1427
254	205595660042_R05C01	Zheng 16377
255	208140740105_R08C02	Zhongliao 606
256	205595660042_R12C01	Zhoudou 28

Table S2. Salt damage symptoms and salt tolerance index grades at soybean emergence stage under salt stress (from Liu et al. [41]).

Category	Symptom	Grade	SI [a]	Salt tolerant
Ⅰ	Plant dead, cotyledons were dry (category value is 1)	1	$0.80 \leq SI < 1.00$	Highly salt tolerant
Ⅱ	Plant growth was severely inhibited, cotyledon was not unfolded (category value is 2)	2	$0.60 \leq SI < 0.80$	Salt tolerant
Ⅲ	Plant growth was inhibited with shoot apical meristem, but true leaves were not unfolded (category value is 3)	3	$0.40 \leq SI < 0.60$	Moderately salt tolerant
Ⅳ	Plant growth was basically normal, true leaves were not fully expanded (category value is 4)	4	$0.20 \leq SI < 0.40$	Sensitive
Ⅴ	Plant growth was normal, true leaves were fully expanded (category value is 5)	5	$0.00 \leq SI < 0.20$	Highly sensitive

[a] SI is salt tolerance index at emergence stage.

Table S3. Grading criteria and corresponding salt-injury symptoms at the soybean seedling stage under salt stress (from Liu [42]).

Grade	Symptom	Salt tolerant
1	Healthy green leaves, no damage observed	Highly salt tolerant
2	Slight chlorosis, light yellowish color in true leaves	Salt tolerant
3	Moderate chlorosis, chlorosis in trifoliate leaves	Moderately salt tolerant
4	Severe chlorosis, more than 75% of the leaf area showed chlorosis	Sensitive
5	Dead, plants were completely withered	Highly sensitive

Table S4. Accessions were assigned to six subpopulations (Pop 1–6) based on ADMIXTURE analysis at K = 6.

No.	Test ID	Name	Subpopulation
1	208140740105_R01C02	2170409	2
2	205730040054_R01C01	2170425	2
3	208140740105_R04C02	2170427	2
4	205740000164_R10C02	2170432	2
5	208140740006_R09C02	2170436	2
6	208140740105_R05C02	2170469	2
7	208140740086_R02C01	2170471	2
8	208141520015_R11C01	2170475	3
9	208140740077_R10C01	2170477	3
10	208140740077_R08C01	2261363	6
11	208141520012_R09C01	2261372	6
12	208140740077_R07C02	2261453	2
13	208140740077_R01C02	2270208	3
14	208140740006_R03C02	2270961	3
15	208141520012_R11C02	2271072	6
16	208140740086_R11C01	2271360	3
17	208140740077_R07C01	2271387	3
18	208140740077_R04C02	2271465	2
19	208141520015_R05C02	2271510	4
20	208141520015_R03C02	2271516	3
21	208141520012_R01C01	2271627	4
22	208140740086_R10C01	2271693	6
23	208141520012_R04C02	2271714	6
24	208140740108_R01C02	20P5	2
25	208141520015_R11C02	20SQ041	5
26	208140740077_R10C02	20SQ301	5
27	208141520012_R03C02	21MD5056-060	4
28	208141520015_R02C01	21MD7043	1
29	208140740105_R03C01	21MD8019-021	4
30	205740000001_R05C01	21P009-012	4
31	205740000001_R07C02	21P029-032	6
32	205740000001_R08C02	21P037-040	2
33	208140740105_R06C02	21P041-044	6
34	205740000001_R09C02	21P049-052	6
35	205740000001_R10C02	21P061-064	6
36	208140740077_R02C01	21P073-076	6
37	205740000001_R11C02	21P081-084	6
38	205740000001_R12C02	21P085-088	4
39	208140740105_R02C01	21P093-096	4
40	208140740006_R12C02	21SQP025-028	2
41	208141520012_R03C01	21SQP049-052	2
42	208140740086_R09C02	21SQJian0004-0006	4
43	208140740010_R03C02	21Jian0004	4

44	208141520012_R08C01	21Jian0005	4
45	208140740086_R07C01	21Jian0009	4
46	208140740006_R10C01	21Jian0014-0015	4
47	208140740105_R10C01	21Jian0017	4
48	205740000164_R09C02	21Jian0019	4
49	208141520015_R10C01	21Jian0031-0033	2
50	208141520012_R05C01	21Jian0034-0036	5
51	208140740006_R01C01	21Jian0040-0042	5
52	208140740006_R12C01	21Jian0043-0045	5
53	205740000001_R03C02	21Jian0058-0060	6
54	208140740086_R12C02	21Jian0064-0066	2
55	205740000001_R04C02	21Jian0094-0096	2
56	208140740006_R06C02	21Jian0103-0105	6
57	205740000001_R05C02	21Jian0151-0153	2
58	205740000001_R06C02	21Jian0172-0174	2
59	205740000001_R11C01	21Jian0175-0177	4
60	208140740006_R07C02	21Jian0181-0183	2
61	208141520015_R09C01	21Jian0184-0186	2
62	208140740086_R03C02	21Jian0196-0198	2
63	208140740077_R11C01	21Jian0211-0213	2
64	208140740105_R11C01	21Jian0214-0216	2
65	205740000001_R02C02	21Jian0229-0231	1
66	205740000001_R09C01	21Jian0253-0255	1
67	208140740006_R11C02	21Jian0262-0264	1
68	208140740105_R02C02	21Jian0268-0270	1
69	208140740105_R06C01	21Jian0277-0278	1
70	208140740108_R01C01	21Jian0447-0449	4
71	205740000001_R10C01	21Jian0509-0511	4
72	208141520012_R11C01	21Jian0518-0520	4
73	205740000001_R03C01	21Jian0524-0526	4
74	205740000164_R08C02	21Jian0533-0535	4
75	208140740105_R12C01	21Jian0566-0568	4
76	205740000164_R07C02	21Jian0593-0595	4
77	205740000164_R06C02	21Jian0596-0598	4
78	208140740010_R04C02	21Jian0704-0706	1
79	208140740105_R03C02	21Jian0779-0781	1
80	208141520015_R08C02	21Jian0788-0790	1
81	208140740086_R06C02	21Jian0800-0802	1
82	208140740077_R09C01	21Jian0806-0808	1
83	208140740086_R09C01	21Jian0812-0814	2
84	205740000164_R04C02	21Jian0821-0823	2
85	208141520015_R03C01	21Jian0848-0850	2
86	208141520015_R04C02	21Jian0920-0922	2
87	208140740086_R05C02	21Jian0941-0943	6
88	208140740077_R03C01	21Jian0944-0946	6
89	208141520015_R06C01	21Jian1007-1009	6

90	208140740086_R11C02	21Jian1016-1018	6
91	208140740077_R11C02	21Jian1019-1021	6
92	208140740077_R01C01	21Jian1070-1072	2
93	208141520015_R05C01	21Jian1109-1111	2
94	208140740105_R07C02	21Jian1148-1150	2
95	205740000001_R07C01	21Jian1169-1171	2
96	205730040054_R02C01	21Jian1178-1180	2
97	208140740006_R06C01	21Jian1184-1186	2
98	208140740105_R12C02	21Jian1187-1189	5
99	208140740105_R11C02	21Jian1193-1195	5
100	205740000001_R08C01	21Jian1196-1198	5
101	208140740105_R04C01	21Jian1205-1207	5
102	208140740006_R05C01	21Jian1231	4
103	205730040054_R05C01	21Jian1246	5
104	208140740077_R05C01	21Jian1249	5
105	208141520015_R12C02	21Jian1250	5
106	208140740006_R08C01	21Jian1306	6
107	208140740086_R05C01	21Jian1307	2
108	208141520012_R06C01	21Jian1348	2
109	208140740006_R07C01	21Jian1349	2
110	208140740077_R08C02	21Jian1352	2
111	208141520012_R06C02	21Jian1361	2
112	208141520012_R12C02	21Jian1384	4
113	208140740077_R12C01	21Jian1386	6
114	208140740086_R02C02	21Jian1398	2
115	208140740077_R12C02	21Jian1404	6
116	208140740105_R07C01	21Jian1405	6
117	208141520012_R07C02	21Jian1407	6
118	208141520015_R10C02	21Jian1409	6
119	208140740086_R01C02	21Jian1414	6
120	208140740105_R10C02	21Jian1424	6
121	208141520012_R02C02	2280037-0039	4
122	208140740077_R03C02	22P333	3
123	208140740086_R03C01	22XX6253	2
124	208141520012_R01C02	22XX70040	3
125	208140740006_R08C02	22XX8001	2
126	208140740077_R06C02	22XXMS2260	2
127	208141520012_R05C02	22XXNMD10025	4
128	208140740045_R02C01	22XXNMD10031	4
129	208141520015_R08C01	22XXNMD6082	2
130	208140740086_R04C02	22XXNMD6127	3
131	208140740006_R02C01	22XXNMD6130	3
132	208140740105_R01C01	22XXNMD6139	3
133	208141520015_R01C01	22XXNMD6145	4
134	208140740006_R04C01	22XXNMD6148	4
135	208140740006_R10C02	22XXNMD7103	4

136	208141520012_R02C01	22XXSQJian058	2
137	208140740086_R10C02	22XXSQJian178	4
138	208140740086_R01C01	22XXJian1091	2
139	208141520015_R02C02	22XXJian1109	2
140	208140740006_R04C02	22XXJian1112	2
141	208140740086_R08C02	22XXJian1268	2
142	208140740077_R05C02	22XXJian1298	2
143	208140740105_R05C01	22XXJian1310	4
144	208141520015_R07C01	22XXJian1322	2
145	208140740006_R09C01	22XXJian1337	4
146	208141520012_R08C02	22XXJian1376	3
147	208141520012_R10C01	22Jian0125	3
148	208140740045_R01C01	22Jian1196	4
149	208141520012_R12C01	22Jian1223	4
150	208141520012_R04C01	22Jian1400	2
151	208141520015_R04C01	22Jian1409	2
152	208141520012_R10C02	22Jian1412	2
153	208140740086_R07C02	22Jian1418	2
154	208140740086_R12C01	22Jian1697	1
155	208141520012_R09C02	22Jian1730	3
156	208140740006_R02C02	22Jian3580	2
157	208270070074_R07C01	23J2077-2080	2
158	208270070074_R08C01	23J3205-3207	4
159	208270070074_R11C01	23J3271-3273	4
160	208270070074_R09C01	23J3391-3393	4
161	208270070045_R10C02	23J3415-3417	3
162	208270070045_R08C02	23J3466-3468	5
163	208270070074_R01C01	23J3691-3693	4
164	208270070045_R05C02	23J3811-3813	3
165	208270070074_R02C01	23J3835-3837	4
166	208270070074_R05C01	23J3841-3843	4
167	208270070045_R11C01	23J3898-3900	2
168	208270070045_R04C02	23J3904-3906	2
169	208270070074_R11C02	23J5625-5628	1
170	208270070045_R10C01	23J5649-5652	5
171	208270070045_R11C02	23J5945-5948	4
172	208270070074_R01C02	23J5953-5956	4
173	208270070045_R01C02	23J6004-6006	4
174	208270070074_R10C02	23J6328-6330	3
175	208270070045_R12C01	23J6538-6540	3
176	208270070074_R06C02	23J6541-6543	2
177	208270070045_R07C02	23J6547-6549	4
178	208270070045_R09C02	23J6565-6567	4
179	208270070074_R10C01	23J6574-6576	4
180	208270070074_R05C02	23J6601-6603	4
181	208270070074_R08C02	23J6604-6606	6

182	208270070074_R09C02	23J6820-6822	4
183	208270070074_R12C02	23J6850-6851	3
184	208270070074_R03C02	23J6888	4
185	208270070074_R07C02	23J7001-7004	1
186	208270070074_R12C01	23J7069-7072	3
187	208270070074_R04C02	23J7465-7468	4
188	208270070045_R02C02	23J7921-7924	4
189	208270070045_R06C02	23J7929-7932	4
190	208270070074_R03C01	23MD049-051	1
191	208270070074_R02C02	23MD061-063	6
192	208270070074_R04C01	23MD121-123	4
193	208270070045_R12C02	23MD175-177	1
194	208270070045_R03C02	23MD724-726	4
195	208270070074_R06C01	23MD730-732	4
196	208140740010_R10C01	782-1	2
197	208140740045_R03C01	860-6	2
198	208140740010_R05C01	a+a'Yaji	4
199	208140740108_R04C02	aYaji	2
200	208140740059_R08C02	PI 416937	4
201	208140740045_R09C01	Cangdou 15	4
202	203568950003_R01C01	Fendou 78	4
203	208140740105_R09C02	Fendou 95	2
204	208140740010_R03C01	Gong552	2
205	205740000133_R08C01	Handou 13	4
206	205740000133_R09C01	Handou 14	6
207	208140740108_R02C01	Huangdou 12	4
208	208140740108_R02C02	Jinong 58	2
209	208140740059_R09C02	Jiyu 397	2
210	208140740108_R10C02	Jiyu 548	2
211	208140740010_R11C01	JiHJ116	4
212	203757450074_R02C01	Jidou 12	4
213	208140740045_R04C02	Jinhuang 10	4
214	205740000133_R02C02	Jinke 5	2
215	203757450034_R05C01	Jinyi 51	4
216	208140740108_R07C01	Kedou 35	4
217	208141520015_R09C02	Kefeng 29	4
218	205595660042_R11C02	Longzhonghuang 601	4
219	208140740010_R02C02	Mengkedou 12	2
220	208140740010_R01C01	NinghuangLD89	1
221	205740000133_R04C01	Ningjingdou 7	2
222	203568950003_R04C01	Qihuang 34	3
223	208140740086_R04C01	Qihuang 42	4
224	205600730158_R08C01	Shanning 17	4
225	205740000133_R07C02	Shanning 21	4
226	208140740108_R12C01	Shanning 27	3
227	208140740045_R08C02	Shanning 29	3

228	203568950003_R10C01	Shengdou 10	4
229	208140740108_R11C01	Shengdou 24	4
230	205740000133_R12C01	Shi 885	2
231	203757450034_R12C01	Shidou 17	2
232	203757450122_R03C02	Sudou 13	4
233	205739190117_R06C01	Suike 23	4
234	205730040062_R11C01	Wandou 37	4
235	203757450075_R07C01	Weidou 1897	4
236	203550230076_R02C02	Wuxing 4	4
237	208140740105_R08C01	Xiangfeng 2	4
238	203757450075_R02C02	Xiangfeng 4	4
239	203757420057_R04C01	Xingdou 5	2
240	208141520012_R07C01	Xu9416-6bai	4
241	205739190011_R12C01	Xudou 14	4
242	203581760004_R10C02	Xudou 18	4
243	203581760004_R12C02	Xudou 20	4
244	205739190106_R04C01	Xudou 21	4
245	205740000074_R12C01	Xudou 22	4
246	205740000022_R11C02	Xudou 23	4
247	205740000074_R01C02	Xudou 24	4
248	205730040028_R09C02	Xudou 25	4
249	205600730192_R07C01	Xudou 9	4
250	208140740108_R11C02	Yundou 202	6
251	203757450120_R01C01	Yundou 1	4
252	203757450074_R07C02	Zheng 1311	4
253	208140740077_R04C01	Zheng 1427	4
254	205595660042_R05C01	Zheng 16377	4
255	208140740105_R08C02	Zhongliao 606	2
256	205595660042_R12C01	Zhoudou 28	4

Table S5. SNPs significantly associated with salt-tolerance-related traits.

SNP	Associated traits	Chr.	Position	Method	Max_-$\log_{10}(P)$	Mean_Effect	Assigned QTL interval
Gm01_14874637	SI-INT[a]	1	14874637	FarmCPU, GLM, SUPER	6.32	0.2516	Gm01_14874637-14927241
Gm01_14927241	SI-INT	1	14927241	FarmCPU, GLM, SUPER	6.09	0.1034	Gm01_14874637-14927241
Gm01_17436477	SI-INT	1	17436477	FarmCPU, GLM, SUPER	6.32	0.2291	Gm01_17436477
Gm01_18506780	SI-INT	1	18506780	FarmCPU, GLM, SUPER	6.32	0.2291	Gm01_18506780
Gm01_19151980	SI-INT	1	19151980	FarmCPU, GLM, SUPER	6.32	0.2291	Gm01_19151980
Gm02_3640916	SI-INT	2	3640916	BLINK, FarmCPU, GLM, SUPER	6.78	-0.2796	Gm02_3640916-3649811
Gm02_3649811	SI-INT	2	3649811	BLINK, FarmCPU, GLM, SUPER	7.82	0.1932	Gm02_3640916-3649811
Gm03_38525925	STG-SSB[b]	3	38525925	CMLM, GLM, MLM	4.41	0.1691	Gm03_38525925-38815291
Gm03_38551345	STG-SSB	3	38551345	CMLM, GLM, MLM	4.49	0.1734	Gm03_38525925-38815291
Gm03_38571249	STG-SSB	3	38571249	CMLM, GLM, MLM	5.19	0.1876	Gm03_38525925-38815291
Gm03_38601323	STG-SSB	3	38601323	BLINK, CMLM, FarmCPU, GLM, MLM, MLMM	8.64	0.2038	Gm03_38525925-38815291
Gm03_38604274	STG-SSB	3	38604274	CMLM, GLM, MLM	7.72	0.2442	Gm03_38525925-38815291
Gm03_38630798	STG-SSB	3	38630798	CMLM, GLM, MLM	8.03	0.2544	Gm03_38525925-38815291
Gm03_38649937	STG-SSB	3	38649937	CMLM, GLM, MLM	7.83	0.2411	Gm03_38525925-38815291
Gm03_38669600	STG-SSB	3	38669600	CMLM, GLM, MLM	8.03	0.2544	Gm03_38525925-38815291
Gm03_38688580	STG-SSB	3	38688580	BLINK, CMLM, FarmCPU, GLM, MLM	9.08	0.3276	Gm03_38525925-38815291
Gm03_38695836	STG-SSB	3	38695836	CMLM, GLM, MLM	5.01	0.2077	Gm03_38525925-38815291
Gm03_38703846	STG-SSB	3	38703846	CMLM, GLM, MLM	6.43	0.1981	Gm03_38525925-38815291
Gm03_38721090	STG-SSB	3	38721090	CMLM, GLM, MLM	6.03	0.1899	Gm03_38525925-38815291
Gm03_38726519	STG-SSB	3	38726519	CMLM, GLM, MLM	6.56	0.1977	Gm03_38525925-38815291
Gm03_38738661	STG-SSB	3	38738661	CMLM, GLM, MLM	6.56	0.1977	Gm03_38525925-38815291
Gm03_38749251	STG-SSB	3	38749251	CMLM, GLM, MLM	5.09	0.2102	Gm03_38525925-38815291
Gm03_38757124	STG-SSB	3	38757124	CMLM, GLM, MLM	5.27	0.2079	Gm03_38525925-38815291
Gm03_38762864	STG-SSB	3	38762864	CMLM, GLM, MLM	5.68	0.1807	Gm03_38525925-38815291

Gm03_38765053	STG-SSB	3	38765053	CMLM, GLM, MLM	4.97	0.1909	Gm03_38525925-38815291
Gm03_38812538	STG-SSB	3	38812538	CMLM, GLM, MLM	5.64	0.1754	Gm03_38525925-38815291
Gm03_38815291	STG-SSB	3	38815291	CMLM, GLM, MLM	6.04	0.1841	Gm03_38525925-38815291
Gm04_46793849	ST-INT[c]	4	46793849	BLINK, CMLM, FarmCPU, GLM, MLM, MLMM, SUPER	5.70	0.3973	Gm04_46793849
Gm05_35273972	STG-SIB[d]	5	35273972	CMLM, MLM, SUPER	4.39	-0.1480	Gm05_35273972-35303385
Gm05_35303385	STG-SIB	5	35303385	CMLM, MLM, MLMM, SUPER	4.50	-0.1462	Gm05_35273972-35303385
Gm07_6279424	ST-INT	7	6279424	BLINK, FarmCPU, GLM, MLMM	4.37	-0.0018	Gm07_6279424
Gm07_7238076	SI-INT	7	7238076	FarmCPU, GLM, SUPER	6.32	0.2610	Gm07_7238076-7250804
Gm07_7250804	SI-INT	7	7250804	FarmCPU, GLM, SUPER	5.90	0.2343	Gm07_7238076-7250804
Gm09_5191652	ST-INT	9	5191652	BLINK, FarmCPU, GLM, SUPER	6.95	-0.2236	Gm09_5191652
Gm10_3521576	SI-INT	10	3521576	GLM, MLMM, SUPER	4.84	0.3497	Gm10_3521576-3614887
Gm10_3592130	SI-INT	10	3592130	GLM, MLMM, SUPER	4.73	0.3530	Gm10_3521576-3614887
Gm10_3614887	SI-INT	10	3614887	GLM, MLMM, SUPER	4.84	0.3497	Gm10_3521576-3614887
Gm10_38386826	STG-SSB	10	38386826	CMLM, GLM, MLM	5.72	-0.2197	Gm10_38386826-38472859
Gm10_38391181	STG-SSB	10	38391181	CMLM, GLM, MLM	5.47	-0.2129	Gm10_38386826-38472859
Gm10_38398774	STG-SSB	10	38398774	BLINK, CMLM, GLM, MLM	6.01	-0.0092	Gm10_38386826-38472859
Gm10_38403852	STG-SSB	10	38403852	CMLM, GLM, MLM	5.79	-0.2159	Gm10_38386826-38472859
Gm10_38405966	STG-SSB	10	38405966	CMLM, GLM, MLM	5.79	-0.2159	Gm10_38386826-38472859
Gm10_38410919	STG-SSB	10	38410919	CMLM, GLM, MLM	5.20	-0.2045	Gm10_38386826-38472859
Gm10_38418589	STG-SSB	10	38418589	CMLM, GLM, MLM	5.79	-0.2159	Gm10_38386826-38472859
Gm10_38420723	STG-SSB	10	38420723	CMLM, GLM, MLM	5.79	-0.2159	Gm10_38386826-38472859
Gm10_38429001	STG-SSB	10	38429001	CMLM, GLM, MLM	5.79	-0.2159	Gm10_38386826-38472859
Gm10_38434853	STG-SSB	10	38434853	CMLM, GLM, MLM	5.79	-0.2159	Gm10_38386826-38472859
Gm10_38447543	STG-SSB	10	38447543	CMLM, GLM, MLM	5.79	-0.2159	Gm10_38386826-38472859
Gm10_38450905	STG-SSB	10	38450905	CMLM, GLM, MLM	5.79	-0.2159	Gm10_38386826-38472859
Gm10_38456322	STG-SSB	10	38456322	CMLM, GLM, MLM	5.91	-0.2200	Gm10_38386826-38472859
Gm10_38464772	STG-SSB	10	38464772	CMLM, GLM, MLM	5.79	-0.2159	Gm10_38386826-38472859

Gm10_38467316	STG-SSB	10	38467316	CMLM, GLM, MLM	5.58	-0.2210	Gm10_38386826-38472859
Gm10_38472859	STG-SSB	10	38472859	CMLM, GLM, MLM	5.79	-0.2159	Gm10_38386826-38472859
Gm11_2844635	SI-INT	11	2844635	CMLM, MLM, MLMM	4.53	0.3322	Gm11_2844635
Gm11_34520086	STG-SSB	11	34520086	CMLM, GLM, MLM	4.67	0.1784	Gm11_34520086-34522630
Gm11_34522630	STG-SSB	11	34522630	CMLM, GLM, MLM	4.81	0.1848	Gm11_34520086-34522630
Gm12_11112335	STG-SIB	12	11112335	GLM, MLMM, SUPER	5.18	0.2003	Gm12_11112335
Gm13_35700100	ST-INT	13	35700100	BLINK, CMLM, FarmCPU, GLM, MLM, MLMM	4.35	0.4779	Gm13_35700100
Gm14_6728116	SI-INT	14	6728116	BLINK, GLM, SUPER	4.86	0.4184	Gm14_6728116
Gm15_15831968	STG-SIB	15	15831968	BLINK, CMLM, FarmCPU, GLM, MLM, MLMM, SUPER	5.74	0.2379	Gm15_15831968

[a] SI-INT is salt tolerance index after inverse normal transformation at emergence stage; [b] STG-SSB is salt tolerance grades following binary conversion at seedling stage; [c] ST-INT is salt tolerance coefficient after inverse normal transformation at emergence stage; [d] STG-SIB is salt tolerance index grades after binary conversion at emergence stage.

Table S6. Previously reported stress-tolerance genes located near QTLs identified in this study.

QTL	Reported stress-tolerance gene	Physical distance	Biological function	Reference
qST-INT-13	*GmbZIP132*	0.87 Mb	encodes a bZIP transcription factor induced by salt and drought that positively regulates salt tolerance at seed germination	[46]
qST-INT-13	*GmBIN2*	1.39 Mb	encodes a GSK3-type kinase induced by salt and drought and a positive regulator of stress tolerance	[47]
qSI-INT-09	*GmPHD6*	1.70 Mb	overexpression in soybean hairy roots enhances salt tolerance while knockout increases sensitivity	[48]
qSI-INT-10	*GmWRKY54*	2.54 Mb	encodes a WRKY transcription factor that improves drought tolerance through ABA and Ca^{2+} signaling pathways	[49]
qSI-INT-10	*GmERF75*	2.14 Mb	encodes an ERF transcription factor that enhances osmotic stress tolerance via ABA signaling	[50]
qSI-INT-14	*GsPRX*	0.76 Mb	involved in the oxidative stress response	[51]
qSTG-SSB-03	*GmSALT3/GmCHX1*	192.00 kb	seedling-stage salt tolerance gene	[23,24]
